# Shape transitions of red blood cell under oscillatory flows in microchannels

**DOI:** 10.1063/5.0278720

**Published:** 2025-08-11

**Authors:** Lahcen Akerkouch, Trung Bao Le

**Affiliations:** Department of Civil, Construction, and Environmental Engineering North Dakota State University, 1410 14th N, Fargo, North Dakota 58102, USA

## Abstract

This paper aims to examine the ability to control a model of red blood cell (RBC) dynamics and the associated extracellular flow patterns in microfluidic channels via oscillatory flows. Our computational approach employs a hybrid continuum–particle coupling, in which the cell membrane and cytosol fluid are modeled using the dissipative particle dynamics method. The blood plasma is modeled as an incompressible fluid via the immersed boundary method. This coupling is novel because it provides an accurate description of RBC dynamics while the extracellular flow patterns around the RBCs are also captured in detail. Our coupling methodology is validated with available experimental and computational data in the literature and shows excellent agreement. We explore the controlling regimes by varying the shape of the oscillatory flow waveform at the channel inlet. Our simulation results show that a host of RBC morphological dynamics emerges depending on the channel geometry, the incoming flow waveform, and the RBC initial location. Complex dynamics of RBC are induced by the flow waveform. Our results show that the RBC shape is strongly dependent on its initial location. Our results suggest that the controlling of oscillatory flows can be used to induce specific morphological shapes of RBCs and the surrounding fluid patterns in bio-engineering applications.

## I. INTRODUCTION

Extensive research has been conducted in the past few decades on the morphological change of red blood cells (RBCs) in fluid flows due to their importance in blood pathology.^1–3^ It has been shown that the response of the RBC membrane to blood plasma dynamics can affect the overall patterns of microvascular blood flows.^4–8^ Despite a substantial body of literature, the dynamics of RBCs remain a significant challenge to be studied due to the complexity of various response modes, which result from the interaction of the suspended cellular membrane with shear flow.^9^ Several factors can affect the dynamics of RBCs, such as stiffness of the membrane,^10^ shear rate $\left(\bar{\dot{\gamma }}\right)$,^11,12^ and viscosity contrast (*λ*) between the blood plasma and the cytosol,^12^ among other factors. As a result, the RBC deformation process in shear flow is not well-understood, especially under time-dependent shear rates.^13,14^

In free shear flows with a constant shear rate $\left({\bar{\dot{\gamma }}}_{0}\right)$, the shear strength is the controlling parameter of the RBC dynamics, in which the shape of RBCs becomes increasingly complex (more lobes) as the shear rate increases.^15^ In particular, for the range of shear rate $\left({\bar{\dot{\gamma }}}_{0}\right)$ from 10 to 2000 s^−1^, the dynamics of RBCs can be classified into three main regions:^11^ (i) tumbling at weak shear rate $\left({\bar{\dot{\gamma }}}_{0}<10\;s^{-1}\right)$; (ii) circular/elliptical stomatocytes $\left(10\;s^{-1}<{\bar{\dot{\gamma }}}_{0}<400\;s^{-1}\right)$; and (iii) multilobes $\left(400\;s^{-1}<{\bar{\dot{\gamma }}}_{0}<2000\;s^{-1}\right)$. In the tumbling region, the RBC deformation is minimal and reversible, which allows the RBCs to maintain their biconcave discoid shape. As the shear rate increases to 400 s^−1^, the percentage of discocytes decreases and stomatocytes start to dominate. The rolling and tumbling stomatocytes appear at ${\bar{\dot{\gamma }}}_{0}=150$ and 250 s^−1^, respectively. This pattern persists up until ${\bar{\dot{\gamma }}}_{0}=400\;s^{-1}$ when the stomatocytes assume a shape with an elliptical rim. In the range $400\;s^{-1}<{\bar{\dot{\gamma }}}_{0}<2000\;s^{-1}$, RBCs with large lobes on their surface, which are referred to as trilobes or hexalobes, emerge.

Studies of RBC dynamics in microchannels have shown that the RBC can transition from its biconcave discoid shape to different morphologies^4,16,17^ under specific combinations (state diagram) of viscosity contrast, shear rate (the capillary number—*C*_*a*_),^18^ and channel confinement (*χ*).^6,12,17^ The state diagram has revealed two main categories of RBC morphological shapes: (i) symmetrical and (ii) asymmetrical types.^19–21^ The simple type contains three modes:^22^ (a) bullet, (b) croissant (in rectangular channels), and (c) parachute (in circular channels) shapes, while the complex type includes^6,23^ (a) slipper; (b) multilobes; (c) trilobes; and (d) hexalobes shapes. The shape transition in the simple type has been shown to reach a terminal shape (bullet, croissant, or parachute). However, it is still not fully clear whether or not the complex shapes are stable or they are just transient states.^16,24^ In the complex shape mode,^23^ the shape transition mostly depends on the flow lag, which is the difference between the translation velocity of the RBC and the velocity of blood plasma. In brief, it is unclear how the complex shapes emerge from the biconcave discoid shape.

The following two shapes are the most frequently observed:^4^ (i) the croissant shape (symmetrical) and (ii) the slipper shape (asymmetrical). In particular, the slipper shape is characterized by the tank-treading motion of the cell membrane, which is essentially a self-rotation of the membrane around its center of mass during the RBC propagation.^4,25^ Experimental and computational studies have shown that these morphological shapes might result in distinct flow structures of blood plasma in the vicinity of the RBC.^4^ For instance, there exists a closed vortex downstream of the RBC when the slipper shape emerges.^26^ Such a vortex is absent during the croissant shape. To the best of our knowledge, there has been no systematic effort to understand the emergence of the extracellular flow patterns as the morphological shape of the RBC changes.

Recently, oscillatory flow (time-dependent shear rate—${\bar{\dot{\gamma }}}_{f}$) has been shown to be a promising technique for cell separation. Because cell deformation is irreversible under time-dependent shear rates,^13,27^ oscillatory flows have been suggested to sort RBCs based on their size and deformability.^14^ Oscillatory flows can reduce the required travel distance of cells because they induce the lateral migration of cells in a short axial distance. This feature simplifies the design of microfluidic channels and thus improves the cell separation process.^28^ However, it is unclear on the process of morphological transition as the RBC responds to the time-dependent shear rate $\left({\bar{\dot{\gamma }}}_{f}\right)$ during this lateral migration. Therefore, it is necessary to investigate this process in detail.

In this work, we extended our hybrid continuum–particle simulation methodology^29^ to study the response of the RBC to time-dependent shear rates. The main development is the inclusion of cytosol fluid dynamics within the intracellular space, which provides a complete description of RBC dynamics. Our paper is organized as follows: first, a brief description of the numerical methods for simulating the blood plasma and the RBC is presented; second, the obtained RBC dynamics are validated with experimental data under (i) stretching force, (ii) constant shear rates (croissant and slipper shapes), and (iii) oscillatory shear rates; third, we perform a parametric study where the shear rate waveform, the peak flow rate, and the initial position of the RBC are varied to induce a host of RBC morphological changes; and finally, the relationship between the RBC’s shape and the extracellular flow patterns is reported as a basis for cell manipulation in future applications.

## II. METHODOLOGY

### A. Problem formulation

**FIG. 1.**
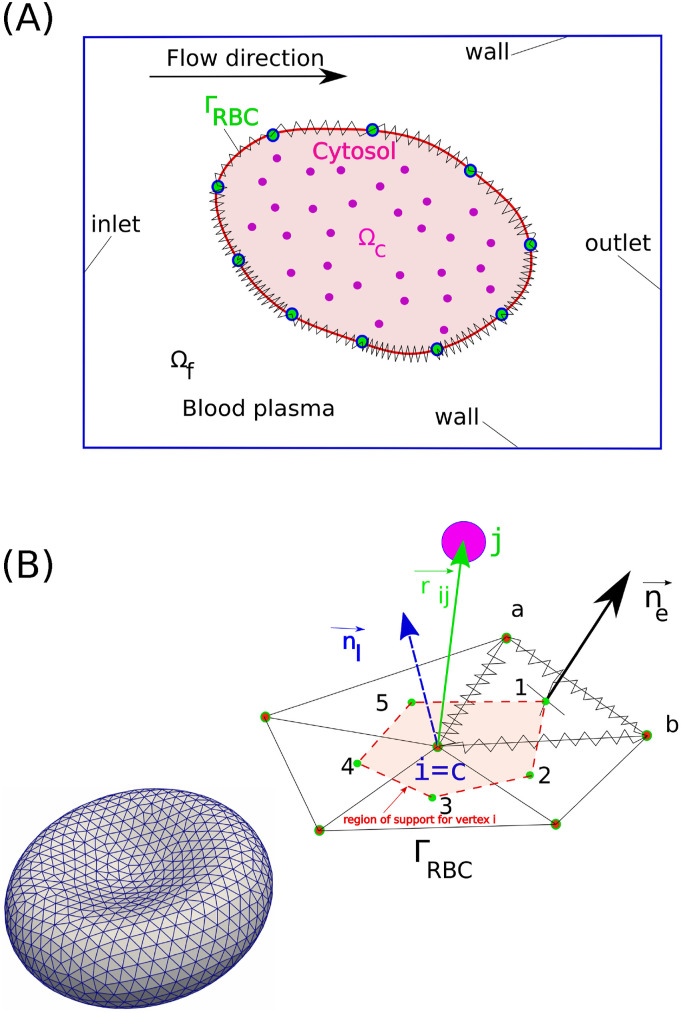
Conceptual framework of the numerical methods. (a) The definitions of the computational domain including the mechanical components of the red blood cells (RBC): the blood plasma (Ω_*f*_); the cell membrane (Γ_*RBC*_); and the cytosol fluid (Ω_*c*_). (b) The triangulated surface that represents Γ_*RBC*_ including the number of vertices (*N*_*v*_) and the number of elements (*N*_*e*_). The schematic of the escaped dissipative particle dynamics (DPD) particle (*j*th-red) from the cytosol fluid outside of the triangulated membrane. Vector ***r***_*ij*_ is the position vector between the membrane vertex *i* = *c* and the escaped cytosol particle *j*th. Here, ***n***_*e*_ is the normal vector of the element **e**. The normal vector (***n***_*i*_) at the vertex *i* = *c* is computed as the averaged value of normal vectors in the adjacent elements ***n***_*e*_ in the region of support. The region of support is defined as the area (dashed line—*A*_*s*_ = *∑*_*e*_*A*_*e*_/3) connecting the centroid of the elements e = 1, 2, 3, 4, and 5.

The red blood cell (RBC) is considered as a deformable body, which is immersed in the fluid plasma Ω_*f*_, as shown in Fig. 1(a). The RBC consists of two components: (i) the cellular membrane (Γ_*RBC*_) and (ii) the cytosol fluid (Ω_*c*_). The outer surface of the RBC’s membrane (Γ_*RBC*_) is the interface between the RBC and the fluid plasma, which is considered as a Newtonian incompressible fluid. Inside the RBC, the cytosol fluid occupies the inner part of the domain Ω_*c*_. The viscosity of the cytosol fluid is different from the one of the plasma fluid (viscosity contrast- $\frac{\nu_{c}}{\nu_{f}}\neq 1$). The RBC deforms under the loading from the fluid domain Ω_*f*_ onto the interface Γ_*RBC*_. In our hybrid continuum–particle approach, different numerical methods are used to model the dynamics of Γ_*RBC*_, Ω_*c*_, and Ω_*f*_, as explained in the following sections.

### B. The idealized shape of the RBC—Γ_*RBC*_

The idealized shape of the RBC membrane is given by a set of points (i) with coordinates (*x*_*i*_, *y*_*i*_, *z*_*i*_) in a 3*D* space with the analytical equation,^30^ as shown in Fig. 1(b),

$$z_{i}=\pm D_{0}\sqrt{1-\frac{4\left(x_{i}^{2}+y_{i}^{2}\right)}{D_{0}^{2}}}\left[a_{0}+a_{1}\frac{x_{i}^{2}+y_{i}^{2}}{D_{0}^{2}}+a_{2}\frac{{\left(x_{i}^{2}+y_{i}^{2}\right)}^{2}}{D_{0}^{4}}\right];$$ (1)

the parameters are chosen in this work as *D*_0_ = 7.82 *µ*m (the equilibrium diameter), *a*_0_ = 0.005 18, *a*_1_ = 2.0026, and *a*_2_ = −4.491. Note that the idealized shape will be used as the initial shape of the RBC membrane for all simulation cases. The membrane mechanics that govern the cellular deformation under loadings will be described in the following sections.

### C. RBC membrane model—Γ_*RBC*_

As the idealized surface of the RBC membrane is known precisely according to Eq. (1), a triangulation procedure is carried out to mimic the distribution of the spectrin links on the membrane as edges of each triangular element (links),^30^ as shown in Fig. 1(b). A network of non-linear springs is generated for each edge to model the dynamics of the spectrin links,^29–31^ as shown in Fig. 1. At each vertex *i*, the dynamics of the links are determined by the membrane force $F_{i}^{\mathrm{membrane}}$, which is linked to Helmholtz’s free energy *V*_*i*_ at the same vertex *i* through the following relationship:

$$F_{i}^{\mathrm{membrane}}=-\frac{\partial V_{i}}{\partial r_{i}},$$ (2)

with **r**_**i**_ being the position vector of vertex *i*. The potential *V*({**r**_*i*_}) incorporates the following physical properties of the lipid bilayer: (a) in-plane stretching; (b) bending stiffness; and (c) area and volume conservation,

$$V\left(\left\{r_{i}\right\}\right)=V_{in-plane}+V_{\mathrm{bending}}+V_{\mathrm{area}}+V_{\mathrm{volume}}.$$ (3)

In this work, the model for membrane mechanics follows closely standard dissipative particle dynamics (DPD) methodologies.^30–33^ The details of the respective potentials are listed in Appendix A 1.

#### 1. Membrane elastic properties

As the membrane mechanical response is modeled by Eq. (2), it is necessary to derive its elastic properties since they do not explicitly appear in the governing equations.^34^ Linear analysis^30,33^ on the non-linear spring network leads to a relationship between the macroscopic elastic properties (shear, area-compression, and Young’s moduli) and the model parameters. The derived linear shear modulus (*μ*_0_), the linear area-compression modulus (*K*), and Young’s modulus (*Y*) are shown in Appendix A 1 b. In addition, the model bending coefficient *k*_*b*_ and the macroscopic membrane bending rigidity *k*_*c*_^30,33,35^ are related as $k_{b}=\frac{2k_{c}}{\sqrt{3}}$.

#### 2. Cellular membrane/cytoskeleton self-interaction

**FIG. 2.**
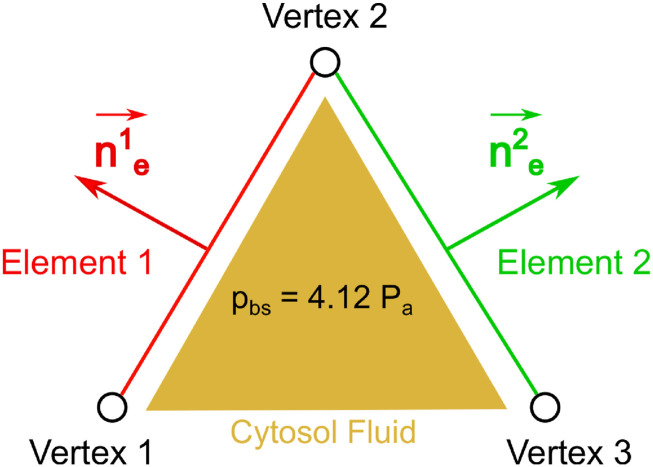
Regularization scheme for the cytoskeletal self-interaction. The intracellular pressure of the cytosol fluid is assumed to increase locally as the two adjacent elements (Element 1 and 2) approach each other. The intracellular pressure is assumed to reach a maximum value of *p*_*bs*_ = 4.12 Pa (the regularization condition). The regularized force **F**^*E*^ [Eq. (4)] results from the increase in the intracellular pressure.

Due to its mechanical properties, the RBC membrane is highly deformable and it can come into contact with its own surface if the external stretching force is sufficiently large locally. To account for this interaction, the bilayer–cytoskeletal interaction force **F**^*E*^ is incorporated into the total RBC membrane forces.^36^ The membrane thickness of a healthy RBC is typically 10 nm, composed of an outer layer (lipid bilayer) and the inner layer (skeleton).^37^ In this study, **F**^*E*^ is applied when the distance between two elements of Γ_*RBC*_ with opposite normal vectors is less than *d*_*a*_. Note that *d*_*a*_ is a parameter of the model, which plays its role only when the cell membrane comes into contact with itself. In this work, *d*_*a*_ is chosen as 20 times the thickness of the membrane (*d*_*a*_ = 0.2 *µ*m). A penalty approach is adopted to compute **F**^*E*^, which is applied equally to all the vertices (*i* = 1, 2 and 3) for each of the two elements. We assume that the cytoplasmic pressure increases locally as two adjacent elements come significantly closer together. We further assume that the maximum intracellular pressure reaches 4.12 Pa, as shown in Fig. 2. The bilayer–cytoskeletal interaction force is given by

$$F_{i}^{E}=p_{bs}A_{e}n_{e},$$ (4)

with a penalty coefficient for the contact traction of the bilayer–cytoskeletal *p*_*bs*_ = 4.12 Pa. *A*_*e*_ and **n**_**e**_ are the area and the normal vector of the element, respectively.

### D. Modeling the interaction (S) between RBC’s membrane (Γ_*RBC*_) and the cytosol fluid (Ω_*c*_)

The interaction between the membrane and the cytosol fluid leads to the emergence of the overall RBC dynamics.^38^ Differentiating between membrane and cytosol particles is essential for accurately capturing RBC mechanics, as it enables realistic modeling of membrane elasticity, internal viscosity contrast, and deformation behaviors under flow.^39^ This interaction is modeled using the dissipative particles dynamics (DPD method). DPD is a microscopic simulation technique widely used to model the flow of complex fluids. Here, the flow is described as a group of clustered interacting particles according to the Lagrangian approach.^30^ In this work, the cytosol within the RBC is modeled using a set of randomly distributed DPD particles (*N*_*f*_) that fill the internal volume of the cell,^30,31^ as shown in Fig. 1(a). Therefore, both the membrane and the cytosol fluid are considered collections of DPD particles.

Due to the different nature of the interactions, the components of the force of each particle depend on the nature of the particle *i* (either membrane or cytosol particle). In general, each DPD particle *i* interact with surrounding particles *j* (either a membrane or cytosol fluid particle) within a cutoff radius *r*_*c*_ through three pairwise additive forces: (a) the conservative force $F_{ij}^{C}$; (b) the dissipative force $F_{ij}^{D}$; (c) the random force $F_{ij}^{R}$, and the penalty force $F_{ij}^{P}$. The relative position vector between the particles *i* and *j* and related terms are given by **r**_**ij**_ = **r**_**i**_ − **r**_**j**_, the distance *r*_*ij*_ = |**r**_**ij**_|, and the unit vector ${\hat{r}}_{ij}=\frac{r_{\mathrm{ij}}}{r_{ij}}$. In addition, **v**_*i*,*j*_ = **v**_*i*_ − **v**_*j*_ is the relative velocity between the particles *i* and *j* with velocities **v**_*i*_ and **v**_*j*_.

For a DPD particle *i* of the cytosol fluid, the total internal force $F_{i}^{\mathrm{internal}}$ is

$$F_{i}^{\mathrm{internal}}=\underset{j\neq i}{\sum }F_{ij}^{C}+F_{ij}^{D}+F_{ij}^{R}+F_{ij}^{P}.$$ (5)

For membrane particles, the total internal force $F_{i}^{\mathrm{internal}}$ acting on each membrane particle is given by the sum of the membrane force $F_{i}^{\mathrm{membrane}}$, the interaction forces from the surrounding DPD fluid particles (the cytosol), and the bilayer–cytoskeletal interactions force **F**^*E*^,

$$F_{i}^{\mathrm{internal}}=F_{i}^{\mathrm{membrane}}+F_{i}^{E}+\underset{j\neq i}{\sum }F_{ij}^{C}+F_{ij}^{D}+F_{ij}^{R}+F_{ij}^{P}.$$ (6)

The mathematical formulation of the conservative force $F_{ij}^{C}$, the dissipative force $F_{ij}^{D}$, the random force $F_{ij}^{R}$, and the penalty force $F_{ij}^{P}$ for the membrane and the cytosol fluid particles are explained in Appendix A 2.

Physically, the cytosol particles must be contained within the space bounded by the Γ_*RBC*_ surface, as shown in Fig. 1(a), at all times. However, the geometrical shape of Γ_*RBC*_ does not appear explicitly in the formulation of **F**^*C*^, **F**^*D*^, or **F**^*R*^ [Eqs. (A10)–(A18), Appendix A 2 a]. To ensure the containment of cytosol particles, it is required that a penalty force^40^ is used to model a bounce-back reflection of cytosol fluid particles whenever they impact the membrane surface (collision force). This algorithm ensures that the cytosol particles remain within the RBC volume. This impact condition is checked using the dot product between the normal vector at the membrane vertex *i* (**n**_**i**_) and the relative position vector (**r**_**ij**_ = **r**_**j**_ − **r**_**i**_) of the cytosol fluid particle *j* as

$$n_{i}\cdot r_{\mathrm{ij}}=\left\{\begin{array}{cc} <0\; & \;\text{Inside}\;\Omega_{c}\\ >0\; & \;\text{Outside}\;\Omega_{c}. \end{array}\right.$$ (7)

Note that the penalty force is only activated when the cytosol particles impact on Γ_*RBC*_, as illustrated in Fig. 1(b) if |**r**_**ij**_| < *r*_*c*_. The normal vector **n**_**i**_ is defined as the average of the normal vectors of supporting elements **n**_**e**_, which share the same vertex *i*. If the cytosol particle is confirmed to impact Γ_*RBC*_, the penalty force **F**^**P**^(**r**_**ij**_) is activated,

$$F_{ij}^{P}=-\beta_{e}\;n_{i},$$ (8)

where *β*_*e*_ = 500 is the collision force coefficient.

We implemented the modified velocity Verlet algorithm,^41^ which consists of two primary steps. The first step involves determining the new position of the particle *i* (**r**_*i*_) while predicting the velocity $\left(\tilde{v_{i}}\right)$, and the second step involves correcting the velocity by utilizing the computed force (**F**_*i*_) based on the predicted velocity and the new position as follows:

$$\begin{array}{c} r_{i}\left(t+dt\right)=r_{i}\left(t\right)+dtv_{i}\left(t\right)+\frac{1}{2}dt^{2}F_{i}\left(t\right),\\ \tilde{v_{i}}\left(t+dt\right)=v_{i}\left(t\right)+\Lambda dtF_{i}\left(t\right),\\ F_{i}\left(t+dt\right)=F_{i}\left(r_{i}\left(t+dt\right),\tilde{v_{i}}\left(t+dt\right)\right),\\ v_{i}\left(t+dt\right)=v_{i}\left(t\right)+\frac{1}{2}dt\left(F_{i}\left(t\right)+F_{i}\left(t+dt\right)\right), \end{array}$$ (9)

where $\tilde{v_{i}}\left(t+dt\right)$ is the predictive velocity at time *t* + *dt* and Λ is the variable which accounts for the effects of the stochastic processes. The value of Λ is chosen to be the optimal value^41^ Λ = 0.65. Note that the stochastic processes are related to the thermal fluctuations and thus *dt* must be chosen in the order of microseconds so that these processes can be captured accurately by the simulation.

Here, the computed force is the sum of the external and internal forces as $F_{i}=F_{i}^{\mathrm{internal}}+F_{i}^{\mathrm{external}}$. The external force $F_{i}^{\mathrm{external}}$ is either known (applied directly on the cell membrane) or computed from the extracellular fluid flows (fluid-structure interaction), as explained in the following.

### E. Continuum approach for the blood plasma (F)

The blood plasma is considered as an incompressible Newtonian fluid modeled using the incompressible three-dimensional unsteady Navier–Stockes equations, with density *ρ* and kinematic viscosity $\nu =\frac{\mu_{\mathrm{plasma}}}{\rho }$. The governing equations (continuity and momentum) for the domain Ω_*f*_ are read in Cartesian tensor notation as follows (*i* = 1, 2, 3 and repeated indices imply summation):

$$\begin{array}{c} \frac{\partial u_{i}}{\partial x_{i}}=0,\\ \frac{\partial u_{i}}{\partial t}+\frac{\partial \left(u_{i}u_{j}\right)}{\partial x_{j}}=-\frac{\partial p}{\partial x_{i}}+\nu \frac{\partial^{2}u_{i}}{\partial x_{j}\partial x_{j}}. \end{array}$$ (10)

In the above-mentioned equations, *u*_*i*_ is the *i*th component of the velocity vector **u**; *t* is time; *x*_*i*_ is the *i*th spatial coordinate; and *p* is the pressure divided by *ρ*. The characteristic velocity scale is chosen as *U*_0_. The length scale *L*_*s*_ is set to equal 8 *µ*m for all cases. Note that this length scale is chosen to reflect the diameter of the RBC under the equilibrium condition (*L*_*s*_ ≈ *D*_0_). While the problems in the current study consider the dynamics of a RBC in microchannels, our numerical method is generally applicable for a wider range of applications including those with larger shear rates since the all terms of the Navier–Stokes equations are included.

The fluid solver is based on the sharp-interface curvilinear-immersed boundary (CURVIB) method (https://www.osti.gov/biblio/1312901) in a background structured-grid that contains the RBC model.^29^ The computational grid is formulated using the hybrid staggered/non-staggered methodology.^42^ The Navier–Stokes equations are discretized using second-order accurate differencing for all spatial derivatives. The discrete equations are integrated in time via a second-order accurate fractional step method. The momentum equation is solved with an implicit Runge–Kutta solver. The total mass flux of the entire domain is enforced strictly to machine zero to satisfy the incompressibility constraint of the blood plasma. The flexible generalized Minimal residual (*FGMRES*) method with multigrid pre-conditioner is used to solve the Poisson equation, which results from the discrete continuity equation.^43^

The complex surface of Γ_*RBC*_ is handled by the sharp-interface immersed boundary method, which requires only the velocity reconstruction at the immersed surface along the local normal to the body.^43^ The presence of Γ_*RBC*_ is represented by a layer of computational grid points (immersed surface—IB nodes), which is distributed around the surface Γ_*RBC*_. The boundary conditions reconstructed at the IB nodes are the three velocity components, which are reconstructed by interpolation.^44^ In this work, a linear velocity profile along the normal direction of the immersed surface is assumed near RBC’s boundary.^45^ Therefore, the presence of the IB nodes in our algorithm can be considered as an interface where the fluid and the cellular structure exchanges. The CURVIB method used here has been applied and validated in various FSI problems across different biological engineering areas.^46–49^

To enable the continuum–particle coupling of the blood plasma and the cellular structures in our FSI algorithm, the flow imparted loads $\left(F_{i}^{\mathrm{external}}\right)$ must be calculated on Γ_*RBC*_ as required by Eq. (9).

The fluid stress tensor components $\sigma_{ij}^{f}$ are evaluated locally at every blood plasma points using the second-order differencing schemes for the velocity components using the continuum approach,^44^

$$\sigma_{ij}^{f}=-pI+\mu \left(\frac{\partial u_{i}}{\partial x_{j}}+\frac{\partial u_{j}}{\partial x_{i}}\right),$$ (11)

where ***I*** is the unit tensor. The traction vector ***t***_*e*_ = ***σ***^***f***^***n***_*e*_ is calculated at the center of the triangular element *e* [see Fig. 1(b)]. The traction vector at the vertex is interpolated from ***t***_*I*_ using the inverse distance weighting method^44^ from elements in the region of support. Finally, the external force (***F***^*external*^) at the vertex can be determined as

$$F^{\mathrm{external}}=t_{I}A_{s}.$$ (12)

Here, *A*_*s*_ is the total area of the region of support for the vertex, as seen in Fig. 1(b).

### F. Fluid–structure interaction simulation of RBC in flows

In the current work, we employ the *loose coupling* approach^50^ for the fluid–structure interaction. First, the Navier–Stokes equations are solved (the fluid solver F) to compute the flow field (pressure and velocities in Ω_*f*_) at time step *n*. The loading (traction vector) on the cell membrane is computed using Eq. (12). Using the continuity of the stresses, the external loading on Γ_*RBC*_ is then computed to supply for Eq. (9) (the DPD solver—S) to find the deformation and velocity of Γ_*RBC*_. Via the continuity of the velocity on the immersed boundary, ${\left(r_{i},v_{i}\right)}^{n+1}$ are used as boundary conditions on Γ_*RBC*_ in the next time step *n* + 1. In shorthand notation,

$${\left(r_{i},v_{i}\right)}^{n+1}=S\left(F\left({\left(r_{i},v_{i}\right)}^{n}\right)\right).$$ (13)

### G. Computational setups

**FIG. 3.**
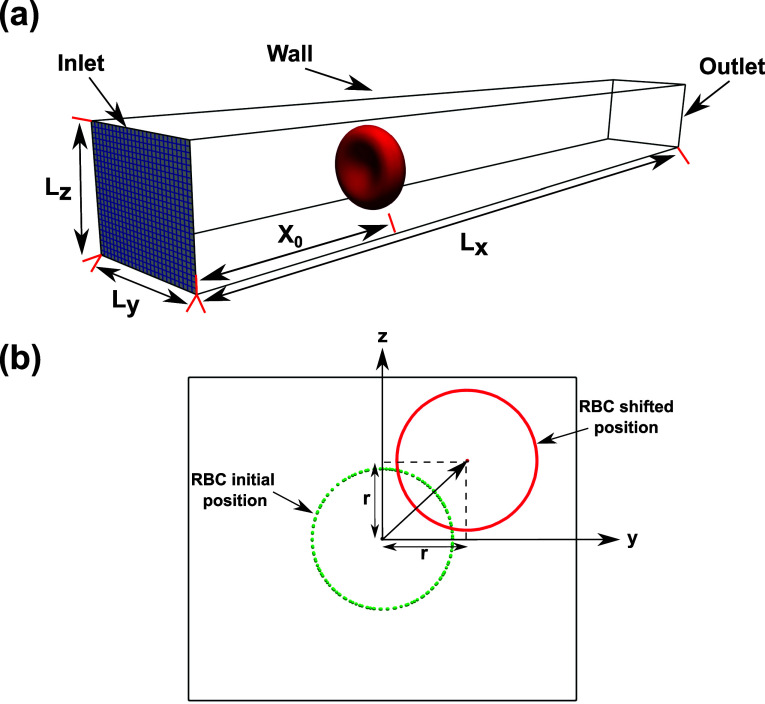
(a) Computational setup for the FSI simulation of a single RBC in a rectangular channel of size *L*_*x*_ × *L*_*y*_ × *L*_*z*_. The inlet plane is shown in blue, which shows uniform grid lines to illustrate the computational mesh. The RBC is placed at an axial distance *x*_0_ from the inlet plane. (b) The sketch of the cross section of the computational domain to illustrate the definition of the radial shift (*r*). The dashed line represents the RBC placed at the channel’s center line. The solid line depicts how the cell is transversely shifted from the cross-sectional center along the bisector of the first quadrant by a radial shift (*r*) in the *y*–*z* plane (Table VI).

Fluid–structure interaction simulations are performed to determine the dynamics of RBC in a confined micro-channel.^4^ The physical properties of the RBC are described in Table I. The corresponding DPD parameters are shown for the corresponding number of vertices *N*_*v*_ in Table II. The computation domain is defined as a rectangular channel containing a single RBC, as illustrated in Fig. 3(a). The dimensions of the domain along the *x*, *y*, and *z* are *L*_*x*_ (the length), *L*_*y*_ (the width), and *L*_*z*_ (the height), respectively. The computational domain is discretized as a structured grid of size *N*_*i*_ × *N*_*j*_ × *N*_*k*_ with the spatial resolution in three directions (*i*, *j*, *k*) are Δ*x* ×Δ*y* ×Δ*z*, respectively. The no-slip condition is applied to all channel walls. The details of the channels used in the simulations are listed in Table III.

**TABLE I.** Physical parameters describing the RBC characteristics.

RBC physical parameters	
RBC diameter (*D*_0_)	7.82 *µ*m
RBC area $\left(A_{0}^{\mathrm{tot}}\right)$	135.0 × 10^−12^ m^2^
RBC volume $\left(V_{0}^{\mathrm{tot}}\right)$	94.0 × 10^−18^ m^3^
Elastic shear modulus (*μ*_0_)	6.3 *µ*N/m
Young’s modulus (*Y*)	18.9 *µ*N/m
Bending rigidity (*k*_*c*_)	3.0 × 10^−19^ J
Membrane viscosity (*η*_*m*_)	22.0 × 10^−3^ Pa s
Boltzmann’s constant (*k*_*B*_)	1.380 649 × 10^−23^ m^2^ kg s^−2^ K^−1^
Temperature (*T*)	298 K

**TABLE II.** Coarse-grained parameters for the RBC membrane model for different numbers of vertices *N*_*v*_. The definitions of the parameters $D_{0}^{M}$, *l*_0_, *l*_max_, *p*, *k*_*p*_, and *θ*_0_ are explained in Appendixes A 3 and A 4. The corresponding values of Young’s modulus, global area, local area, and volume constraints in DPD units are *Y*^*M*^ = 392.5, *k*_*a*_ = 4900, *k*_*d*_ = 100, *k*_*v*_ = 5000, respectively. Other parameters are *α* = 1 and $\eta_{m}^{M}=1.8$.

*N*_*v*_	$D_{0}^{M}$	*l*_0_ (*m*)	*l*_max_ (*m*)	*p* (*m*)	*k*_*p*_ (*Nm*^2^)	*θ*_0_ (deg)
500	8.07	5.5614 × 10^−7^	1.2235 × 10^−6^	1.9933 × 10^−9^	6.6288 × 10^−25^	6.86
1 000	8.07	3.7992 × 10^−7^	8.3582 × 10^−7^	2.9179 × 10^−9^	2.1132 × 10^−25^	4.69
3 000	8.07	2.2818 × 10^−7^	5.0199 × 10^−7^	4.8584 × 10^−9^	4.5783 × 10^−26^	2.82
9 000	8.07	1.3035 × 10^−7^	2.8678 × 10^−7^	8.5044 × 10^−9^	8.5357 × 10^−27^	1.61
24 472	8.26	7.5331 × 10^−8^	1.6573 × 10^−7^	1.4716 × 10^−8^	1.6474 × 10^−27^	0.93

**TABLE III.** Different channel geometries and their associated computational grids to simulate the dynamics of RBC in fluid flows. The channels have rectangular cross sections of size *L*_*x*_, *L*_*y*_, and *L*_*z*_ along the axial, spanwise, and vertical directions, respectively. *N*_*i*_, *N*_*j*_, and *N*_*k*_ are the number of grid points in *x*, *y*, and *z* directions, respectively. *χ* is the channel confinement, which is defined in Sec. II G.

Channel	*L*_*x*_ × *L*_*y*_ × *L*_*z*_ (*μ*m)	*N*_*i*_ × *N*_*j*_ × *N*_*k*_	Δ*x* ×Δ*y* ×Δ*z* (*μ*m)	*χ*
1	90 × 12 × 10	151 × 101 × 101	0.6 × 0.12 × 0.1	0.65
2	80 × 16 × 16	151 × 101 × 101	0.54 × 0.16 × 0.16	0.4
3	80 × 21 × 21	151 × 151 × 151	0.54 × 0.14 × 0.14	0.3
4	90 × 12 × 10	151 × 231 × 231	0.6 × 0.05 × 0.005	0.65

The RBC is located initially at *t* = 0 in an axial distance of *x*_0_ from the inlet. The transverse location of the RBC is placed along the bisector of the first quadrant with a radial shift (*r*). Thus, the transverse coordinates of the RBC are *y*_0_ = *r* and *z*_0_ = *r*, respectively, as shown in Fig. 3(b) (*r* is the radial shift). With this configuration, the RBC confinement is defined as the ratio between the effective RBC diameter $D_{r}=\sqrt{\frac{A_{0}^{\mathrm{tot}}}{\pi }}$ (Table I) and the domain height *L*_*z*_,

$$\chi =\frac{D_{r}}{L_{z}}.$$ (14)

The initial shape of the RBC is first set to be the idealized shape [Eq. (1)] for all simulation cases at the initial time *t* = 0. A short period of relaxation *t*_*relax*_ is allowed for the RBC under no external load (no flows) so that the internal forces of the RBC membrane balance. A uniform flow velocity *U*(*t*) is then applied at the channel inlet at *t* > *t*_*relax*_ to induce RBC’s deformation depending on the controlling strategy. The average shear rate across the channel height is defined as the ratio between the bulk velocity *U*(*t*) and the domain’s height,

$$\bar{\dot{\gamma \left(t\right)}}=\frac{U\left(t\right)}{L_{z}}.$$ (15)

At the channel’s outlet, a fully developed flow (Neumann) condition is imposed at the outlet of the channel.

#### 1. Constant shear rate condition (*I*_0_)

Following the experimental study of Guckenberger *et al.*^4^ (Channel-1, Table III), FSI simulations of the RBC in channel flow with a constant flow rate are carried out with *x*_0_ = 22.5 *µ*m. To highlight the constant flow rate, the notation *I*_0_ is introduced to emphasize this condition. As shown in Table IV, a constant inflow velocity *U*(*t*) = *ψ*_0_ is required at the inlet of the computational domain. Two values of *ψ*_0_ are considered: (*i*) *ψ*_0_ = *U*_3_ = 2 mm s^−1^ and (*ii*) *ψ*_0_ = *U*_4_ = 6 mm s^−1^. In these cases, two values of the radial shift are also investigated: *r*_1_ = 0 and *r*_3_ = 0.7 *µ*m. To simplify the discussions, the numerical values for the bulk velocity *ψ*_0_ will not be explicitly referred to. Instead, only the acronyms (*U*_3_ and *U*_4_) will be used for reasons that will be evident in the subsequent texts.

**TABLE IV.** Summary of the validation cases under constant shear rates (*I*_0_*U*_3_*r*_1_*χ*_1_ and *I*_0_*U*_4_*r*_3_*χ*_1_) and stepwise oscillatory flows (*I*_*s*_*ψ*_1_*r*_1_*χ*_2_, *I*_*s*_*ψ*_2_*r*_1_*χ*_2_, *I*_*s*_*ψ*_3_*r*_1_*χ*_2_ and *I*_*s*_*ψ*_4_*r*_1_*χ*_3_, *I*_*s*_*ψ*_5_*r*_1_*χ*_3_, and *I*_*s*_*ψ*_6_*r*_1_*χ*_3_). The stepwise oscillatory flows with the forward (*ψ*_*f*_) and backward (*ψ*_*b*_) velocities and the forward Capillary number are defined in Sec. II G 2. The maximum shear rate $\left({\bar{\dot{\gamma }}}_{f}\right)$ and the maximum Reynolds number (*Re*_*f*_) are defined in Sec. II G 1. The definition of RBC’s radial shift *r* is shown in Fig. 3(b).

Case	Inflow	*ψ*_*f*_ (mm s^−1^)	*ψ*_*b*_ (mm s^−1^)	${\bar{\dot{\gamma }}}_{f}\left(s^{-1}\right)$	*r* (*μ*m)	*Re*_*f*_	*Ca* ^*f*^
*I*_0_ *U*_3_ *r*_1_ *χ*_1_	*I*_0_	2	⋯	200	0	1.34 × 10^−2^	0.49
*I*_0_ *U*_4_ *r*_3_ *χ*_1_	*I*_0_	6	⋯	600	0.7	4 × 10^−2^	1.47
*I*_*s*_ *ψ*_1_ *r*_1_ *χ*_2_	*I*_*s*_	1.05	−0.27	66	0	7 × 10^−3^	0.1
*I*_*s*_ *ψ*_2_ *r*_1_ *χ*_2_	*I*_*s*_	1.58	−0.39	98	0	1.05 × 10^−2^	0.15
*I*_*s*_ *ψ*_3_ *r*_1_ *χ*_2_	*I*_*s*_	2.1	−0.53	132	0	1.4 × 10^−2^	0.2
*I*_*s*_ *ψ*_4_ *r*_1_ *χ*_3_	*I*_*s*_	1.9	−0.48	119	0	1.27 × 10^−2^	0.1
*I*_*s*_ *ψ*_5_ *r*_1_ *χ*_3_	*I*_*s*_	2.8	−0.7	175	0	1.87 × 10^−2^	0.15
*I*_*s*_ *ψ*_6_ *r*_1_ *χ*_3_	*I*_*s*_	3.7	−0.93	232	0	2.47 × 10^−2^	0.2

Using these notations, the FSI simulation cases are named using the convention for each type of inflow waveform (*I*), the bulk velocity (*U*), the radial shift (*r*), and the channel type, respectively. The first case (*I*_0_*U*_3_*r*_1_*χ*_1_) is configured with $\left(\psi_{0}=U_{3}=2\;\;mm\;s^{-1}\right)$ and *r* = *r*_1_ = 0 *µ*m. The second case (*I*_0_*U*_4_*r*_3_*χ*_1_) is carried out with *ψ*_0_ = *U*_4_ = 6 mm s^−1^ and *r* = *r*_3_ = 0.7 *µ*m. Here, the Reynolds number is defined as $R_{e}=\frac{UL_{s}}{\nu }$. The kinematic fluid viscosity of blood plasma is chosen as $\nu =\frac{\mu_{\mathrm{plasma}}}{\rho }=1.2\times 10^{-6}\;m^{2}/s$. The summary of the parameters for each simulation case is shown in Table IV.

First, *t*_*relax*_ = 10 and 7.0 ms are set for *I*_0_*U*_3_*r*_1_*χ*_1_ (croissant) and *I*_0_*U*_4_*r*_3_*χ*_1_ (slipper) simulations, respectively. After the relaxation period, a linear ramping period is set for each simulation case *t*_*ramp*_ = 30 and 20 ms for *I*_0_*U*_3_*r*_1_*χ*_1_ and *I*_0_*U*_4_*r*_3_*χ*_1_, respectively. During this ramping period, the bulk velocity *U*(*t*) is linearly increased. The value of *U*(*t*) reaches *ψ*_0_ at the end of the ramping period.

#### 2. Stepwise oscillatory flows (*I*_*s*_)

**FIG. 4.**
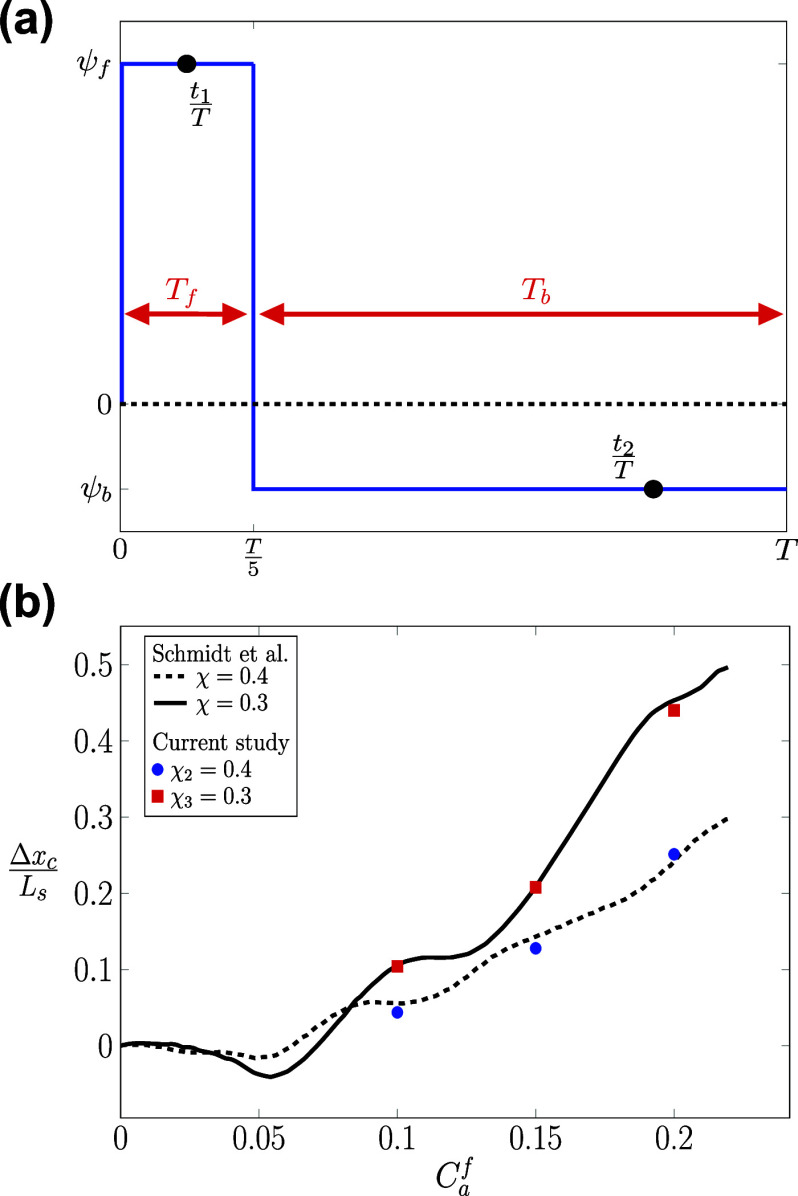
Propulsion step Δ*x*_*c*_ [Eq. (18)] as a function of the forward capillary number *Ca*^*f*^ [Eq. (17)]. The propulsion step Δ*x*_*c*_ is shown in terms of the length scale (*L*_*s*_ = 8 *µ*m). (*a*) The bulk flow waveform of the inflow (*U*(*t*)) has a stepwise shape [see Eq. (16)]. Two-time instances ($\frac{t_{1}}{T}$ and $\frac{t_{2}}{T}$) are shown to exemplify the changes in RBC shapes over time. (b) Three values of *Ca*^*f*^ = 0.1, 0.15, and 0.2 (red squares and blue circles) are simulated. The computed values of Δ*x*_*c*_ are compared to the study in Ref. 13 (solid lines).

To further validate our FSI model in oscillatory flows, the propulsion of the RBC in square channels is investigated.^13^ Two square channels (Channel-2 and Channel-3 in Table III) with side lengths *L*_*z*_ = 16 and 21 *µ*m are used for the simulations, resulting in confinements *χ*_2_ = 0.4 and *χ*_3_ = 0.3, respectively. The initial location of the RBC is on the channel axis (*x*_0_ = 16 *µ*m, *r* = *r*_1_ = 0). The computational configuration including the grid spacing, RBC surface meshes, and boundary conditions are shown in Fig. 3(a) and Table III. A stepwise asymmetric oscillatory waveform *I*_*s*_ is used with two phases: (i) forward *T*_*f*_ and (ii) backward *T*_*b*_ periods $\left(\frac{T_{b}}{T_{f}}=4\right)$, as shown in Fig. 4(a). The velocities during the forward and backward phases are *ψ*_*f*_ and $\psi_{b}\;\left(-\frac{\psi_{f}}{\psi_{b}}=4\right)$, respectively. The formula for the waveform is defined as

$$U\left(t\right)=\left\{\begin{array}{cc} \psi_{f}\; & \;\;\text{for}\;\;0\leq t<\;\frac{T}{5},\\ \psi_{b}\; & \;\;\text{for}\;\;\frac{T}{5}\leq t<T. \end{array}\right.$$ (16)

Following this formula, the flow has a forward phase (*ψ*_*f*_ > 0) and a backward flow phase (*ψ*_*b*_ < 0). The maximum shear rate is defined as $\bar{\dot{\gamma_{f}}}=\frac{\psi_{f}}{L_{z}}$.

The Capillary number *Ca*^*f*^ in the forward flow phase is given as

$$Ca^{f}=\frac{4\psi_{f}L_{s}t_{R}}{L_{z}^{2}},$$ (17)

with $t_{R}=\frac{L_{s}\mu_{\mathrm{plasma}}}{2\mu_{0}}$ and *μ*_0_ is the shear elastic modulus described in Table I.

There are six values of *ψ*_*f*_ examined as *ψ*_1_ = 1.05, *ψ*_2_ = 1.58, *ψ*_3_ = 2.1, *ψ*_4_ = 1.9, *ψ*_5_ = 2.8, and *ψ*_6_ = 3.7 mm s^−1^, respectively. Following the naming convention of the simulations, six cases are formed with the respective parameters: *I*_*s*_*ψ*_1_*r*_1_*χ*_2_; *I*_*s*_*ψ*_2_*r*_1_*χ*_2_; *I*_*s*_*ψ*_3_*r*_1_*χ*_2_; and *I*_*s*_*ψ*_4_*r*_1_*χ*_3_, *I*_*s*_*ψ*_5_*r*_1_*χ*_3_, and *I*_*s*_*ψ*_6_*r*_1_*χ*_3_, as shown in Table IV. As the waveform applied is of a stepwise nature, there is no relaxation time taken into consideration for these cases (*t*_*relax*_ = 0).

Under these oscillatory conditions, the axial propulsion step (Δ*x*_*c*_) is recorded at the end of the forward time interval of the asymmetric oscillating flow $\left(t=T_{f}=\frac{T}{5}\right)$, as a function of the forward (peak) capillary number *Ca*^*f*^ for the chosen shear rates.^13^ Thus, Δ*x*_*c*_ is defined as the displacement of the RBC’s centroid (*C*) at the end of the forward phase $\left(t=\frac{T}{5}\right)$,

$$\Delta x_{c}=x_{c}\left(t=\frac{T}{5}\right)-x_{c}\left(t=0\right).$$ (18)

#### 3. Sinusoidal flow simulations

To study the effect of the pulsatile flow on the propulsion and the behavior of the cellular response (morphology changes) of the RBC, we considered time-periodic flow *U*(*t*). The flow time period consists of three separate phases: (*i*) the forward (*T*_*f*_); (*ii*) the resting (*T*_*r*_), and the backward (*T*_*b*_) periods. Following experimental protocols,^14^ the resting period *T*_*r*_ was introduced in the bulk flow waveform to allow the RBC to restore its initial equilibrium shape. The oscillatory frequency is chosen to be *f* = 20 Hz^27^ and thus *T* = *T*_*f*_ + *T*_*r*_ + *T*_*b*_ = 50 ms. The asymmetry of the waveform is adjusted by changing the values of *T*_*f*_, *T*_*r*_, and *T*_*b*_. The formula for the waveform is

$$U\left(t\right)=\left\{\begin{array}{cc} A\sin\left(2\pi \frac{t}{T_{f}}\right)\; & \;\;\text{for}\;\;0\leq t\leq \;T_{f},\\ 0\; & \;\;\text{for}\;\;T_{f}\leq t\leq T_{f}+T_{r},\\ A\sin\left(2\pi \frac{\left(t-T_{f}-T_{r}\right)}{T_{b}}\right)\; & \;\;\text{for}\;\;T_{f}+T_{r}\leq t\leq T. \end{array}\right.$$ (19)

**FIG. 5.**
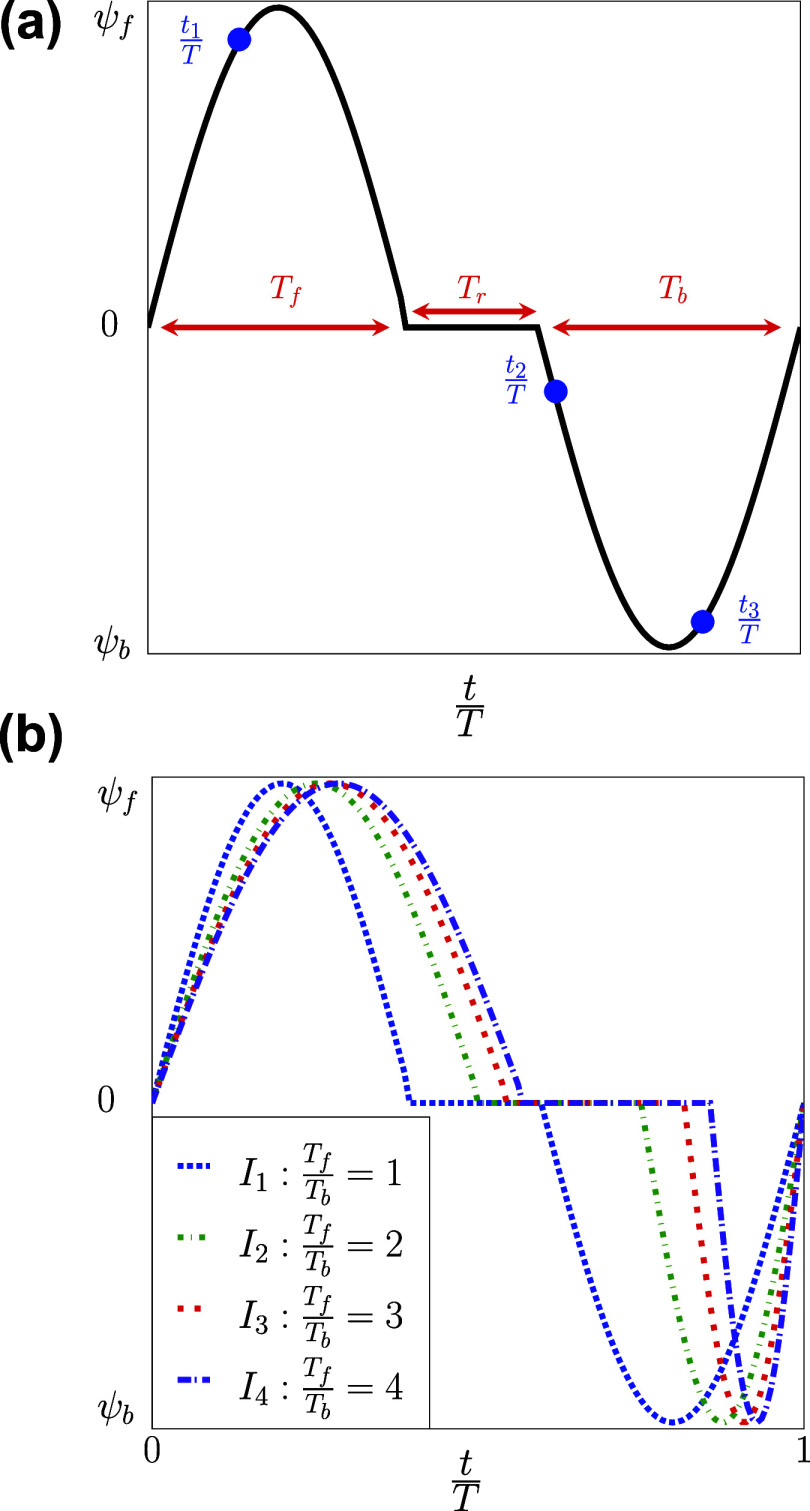
(a) Sinusoidal inflow velocity *U*(*t*) profile with the forward (*T*_*f*_), resting (*T*_*r*_), and backward (*T*_*b*_) time intervals. (b) Four inflow types with different $\frac{T_{f}}{T_{b}}$ rations are considered (see also Table V). The time instances $\frac{t_{1}}{T}$, $\frac{t_{2}}{T}$, and $\frac{t_{3}}{T}$ shown in panel (a) represent instances at which the RBC shapes are recorded. The exact values of the time instances are shown in Fig. 9 and Tables VIII–XI.

The symmetric waveform (*I*_1_) is created with *T*_*f*_ = *T*_*b*_ (completely symmetric). The asymmetric waveforms (*I*_2_, *I*_3_, and *I*_4_) are formed by progressively reducing the period of *T*_*b*_. Four distinct inflow types were generated with symmetry and asymmetric waveforms (*I*_1_, *I*_2_, *I*_3_, and *I*_4_), as seen in Fig. 5 and Tables V and VI. For each of these waveforms, three different velocity magnitudes (*A* = *U*_1_, *U*_2_, and *U*_3_) and three different radial shifts (*r*_1_, *r*_2_, and *r*_3_) are considered, as shown in Table VII. In total, the combinatoric arrangements lead to a total of 36 distinct simulation cases with the notation *I*_*m*_*U*_*n*_*r*_*p*_*χ*_1_ with the corresponding values of the indices *m* = 1, 2, 3, 4; *n* = 1, 2, 3; and *p* = 1, 2, 3. The outline of the simulation cases is shown in Table VII. In addition, the RBC shapes are recorded over a time period of two cycles 2*T* as exemplified in Fig. 5(b), in which the initial location of the RBC is set at *x*_0_ = 22.5 *µ*m. Due to the nature of the sinusoidal waveform applied, there was no relaxation time for all of these cases (*t*_*relax*_ = 0). The centroid’s displacement is monitored continuously as the function of time,

$$\Delta x_{c}\left(t\right)=x_{c}\left(t\right)-x_{c}\left(t=0\right).$$ (20)

**TABLE V.** Controlling parameters of the pulsatile waveforms (*I*_1_, *I*_2_, *I*_3_, and *I*_4_). The waveforms are characterized by the intervals of the forward (*T*_*f*_), rest (*T*_*r*_), and backward (*T*_*b*_) periods. The shapes of the waveforms are shown in Fig. 5.

Time			
Waveforms	*T*_*f*_ (ms)	*T*_*r*_ (ms)	*T*_*b*_ (ms)
*I*_1_	20	10	20
*I*_2_	25	12.5	12.5
*I*_3_	27.3	9	13.7
*I*_4_	28.6	7.1	14.3

**TABLE VI.** Summary of the combinatoric configurations for steady and pulsatile flow simulations. The combination of the waveform type, the forward flow velocity, the radial shift, and the channel confinement results in the simulation configurations of *I*_*m*_*U*_*n*_*r*_*p*_*χ*_*s*_. Here, *m* = 1, 2, 3, and 4; *n* = 1, 2, 3, and 4; *p* = 1, 2, and 3; and *s* = 1, 2, and 3. The profile of the inflow waveforms (*I*_1_, *I*_2_, *I*_3_, and *I*_4_) are shown in Fig. 5. The peak forward flow velocity *U* [Fig. 5(a)] varies from 1 to 6 mm/s. The radial shift of the RBC centroid along the bisector of the *y*–*z* plane at the initial time is defined in Fig. 3(b).

Subscript	Inflow waveform	*ψ*_*f*_ (mm s^−1^)	Radial shift *r* (*μ*m)
1	*I*_1_	*U*_1_ = 1	*r*_1_ = 0
2	*I*_2_	*U*_2_ = 1.5	*r*_2_ = 0.4
3	*I*_3_	*U*_3_ = 2	*r*_3_ = 0.7
4	*I*_4_	*U*_4_ = 6	⋯

**TABLE VII.** Summary of the 36 sinusoidal flow cases in Sec. II G 3 with a confinement of *χ*_1_ = 0.65. Tables (a), (b), (c), or (d) each consist of nine possible combinations between the peak forward flow *U* and the radial shift *r* for each type of waveform *I*_1_, *I*_2_, *I*_3_, and *I*_4_, respectively. The exact numeric value of *U*_1_, *U*_2_, *U*_3_ and *r*_1_, *r*_2_, *r*_3_ are shown in Table VI.

(a)		*r*_1_	*r*_2_	*r*_3_
*I*_1_	*U*_1_	*I*_1_ *U*_1_ *r*_1_ *χ*_1_	*I*_1_ *U*_1_ *r*_2_ *χ*_1_	*I*_1_ *U*_1_ *r*_3_ *χ*_1_
	*U*_2_	*I*_1_ *U*_2_ *r*_1_ *χ*_1_	*I*_1_ *U*_2_ *r*_2_ *χ*_1_	*I*_1_ *U*_2_ *r*_3_ *χ*_1_
	*U*_3_	*I*_1_ *U*_3_ *r*_1_ *χ*_1_	*I*_1_ *U*_3_ *r*_2_ *χ*_1_	*I*_1_ *U*_3_ *r*_3_ *χ*_1_

(b)		*r*_1_	*r*_2_	*r*_3_
*I*_2_	*U*_1_	*I*_2_ *U*_1_ *r*_1_ *χ*_1_	*I*_2_ *U*_1_ *r*_2_ *χ*_1_	*I*_2_ *U*_1_ *r*_3_ *χ*_1_
	*U*_2_	*I*_2_ *U*_2_ *r*_1_ *χ*_1_	*I*_2_ *U*_2_ *r*_2_ *χ*_1_	*I*_2_ *U*_2_ *r*_3_ *χ*_1_
	*U*_3_	*I*_2_ *U*_3_ *r*_1_ *χ*_1_	*I*_2_ *U*_3_ *r*_2_ *χ*_1_	*I*_2_ *U*_3_ *r*_3_ *χ*_1_

(c)		*r*_1_	*r*_2_	*r*_3_
*I*_3_	*U*_1_	*I*_3_ *U*_1_ *r*_1_ *χ*_1_	*I*_3_ *U*_1_ *r*_2_ *χ*_1_	*I*_3_ *U*_1_ *r*_3_ *χ*_1_
	*U*_2_	*I*_3_ *U*_2_ *r*_1_ *χ*_1_	*I*_3_ *U*_2_ *r*_2_ *χ*_1_	*I*_3_ *U*_2_ *r*_3_ *χ*_1_
	*U*_3_	*I*_3_ *U*_3_ *r*_1_ *χ*_1_	*I*_3_ *U*_3_ *r*_2_ *χ*_1_	*I*_3_ *U*_3_ *r*_3_ *χ*_1_

(d)		*r*_1_	*r*_2_	*r*_3_
*I*_4_	*U*_1_	*I*_4_ *U*_1_ *r*_1_ *χ*_1_	*I*_4_ *U*_1_ *r*_2_ *χ*_1_	*I*_4_ *U*_1_ *r*_3_ *χ*_1_
	*U*_2_	*I*_4_ *U*_2_ *r*_1_ *χ*_1_	*I*_4_ *U*_2_ *r*_2_ *χ*_1_	*I*_4_ *U*_2_ *r*_3_ *χ*_1_
	*U*_3_	*I*_4_ *U*_3_ *r*_1_ *χ*_1_	*I*_4_ *U*_3_ *r*_2_ *χ*_1_	*I*_4_ *U*_3_ *r*_3_ *χ*_1_

## III. RESULTS

### A. Coarse-graining validation

**FIG. 6.**
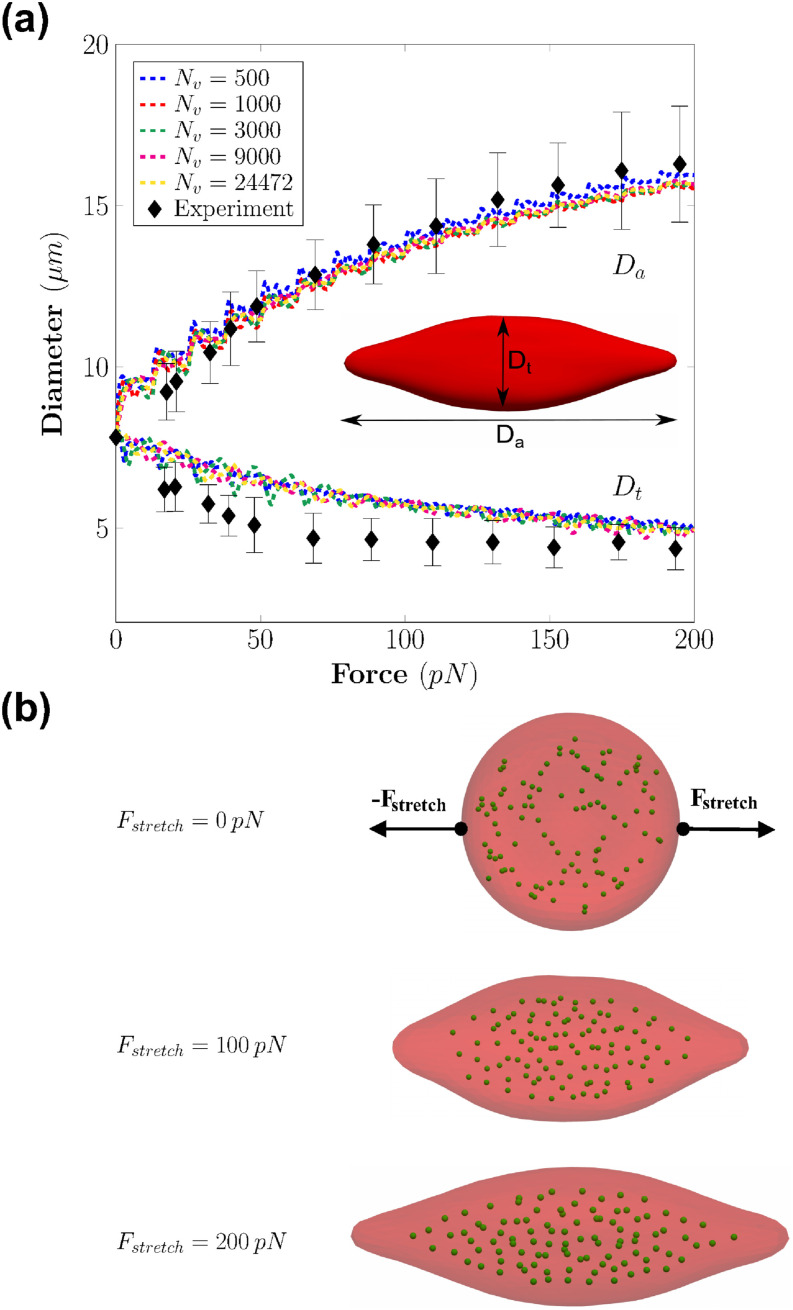
(a) Axial (*D*_*a*_) and transverse (*D*_*t*_) diameters (dashed lines) of the RBC under incremental stretching at different coarse-graining levels (*N*_*v*_) (also see Table II). The experimental data of Ref. 51 are represented by the black diamonds. (b) The deformed shapes of the RBC membrane and the cytoplasm (green particles) under different stretching forces (**F**_**stretch**_).

A stretching test is carried out and aimed to replicate the experimental test of Ref. 51. In our simulations, the parameters used to describe the physical characteristics of the RBC are listed in Table I. Following the coarse-graining procedure, the model parameters for the cell membrane such as the equilibrium length, the persistence length, the spring stiffness, and the spontaneous angle are computed for each value of *N*_*v*_, as in Table II. The cytosol fluid is modeled by a set of particles *N*_*f*_ = 100, placed within the interior volume of the cell membrane, as shown in Fig. 6(b).

In this experiment, two external forces **F**_*stretch*_ and −**F**_*stretch*_ with opposite directions are applied on both sides of the RBC. The magnitude of the force **F**_*stretch*_ is increased in a stepwise manner from 0 to 200 *pN* (a total of 16 steps). The axial diameter (*D*_*a*_) and transverse diameter (*Dt*) are measured for every step. The definitions of *D*_*a*_ and *D*_*t*_ are shown in Fig. 6(a). *D*_*a*_ is calculated as |*x*_max_ − *x*_min_| representing the axial distance between the maximum *x* position, denoted as *x*_max_, and the minimum *x* position, denoted as x_min_ of the membrane vertices. *D*_*t*_ is calculated using the formula $2\times \max_{i=1\;\ldots N_{v}}\sqrt{{\left(y_{i}-c_{y}\right)}^{2}+{\left(z_{i}-c_{z}\right)}^{2}}$, where *c*_*y*_ and *c*_*z*_ represent the *y* and *z* coordinates of the RBC’s center of mass, respectively. The simulations are performed systematically with different RBC surface mesh resolutions by changing the number of vertices (*N*_*v*_) (also see Table II).

The current RBC model accurately replicates the elastic response of the RBC under stretching forces, as revealed by the results of *D*_*a*_ and *D*_*t*_ in Fig. 6. During stretching, the dynamic response of cytosol particles is visible, indicating the coupling between the membrane and the cytosol fluid. The shapes of the RBC under loading conditions agree with ones from experimental data of Ref. 51. The computed values of the axial (*D*_*a*_) and transverse (*D*_*t*_) diameters agree well with the experimental values, as seen in Fig. 6(a). In particular, the values of *D*_*a*_ and *D*_*t*_ are consistent across the different values of *N*_*v*_, which indicates a robust performance of the coarse-graining procedure. There is a disagreement between the simulated results and the experimental value of *D*_*t*_. Examining the shapes of the RBC in the simulations [Fig. 6(b)], it is revealed that the RBC tends to rotate around the stretching direction. This rotation leads to the difference between the experimental and numerical results of *D*_*t*_. In brief, the mechanics of RBC is well-replicated by the computational model across different levels of coarse-graining. Thus, the value of *N*_*v*_ = 1000 is chosen to report the dynamics of the RBC.

### B. Model validation

### 1. *Deformation of the RBC under a constant* *shear rates* $\bar{\dot{\gamma_{0}}}$

**FIG. 7.**
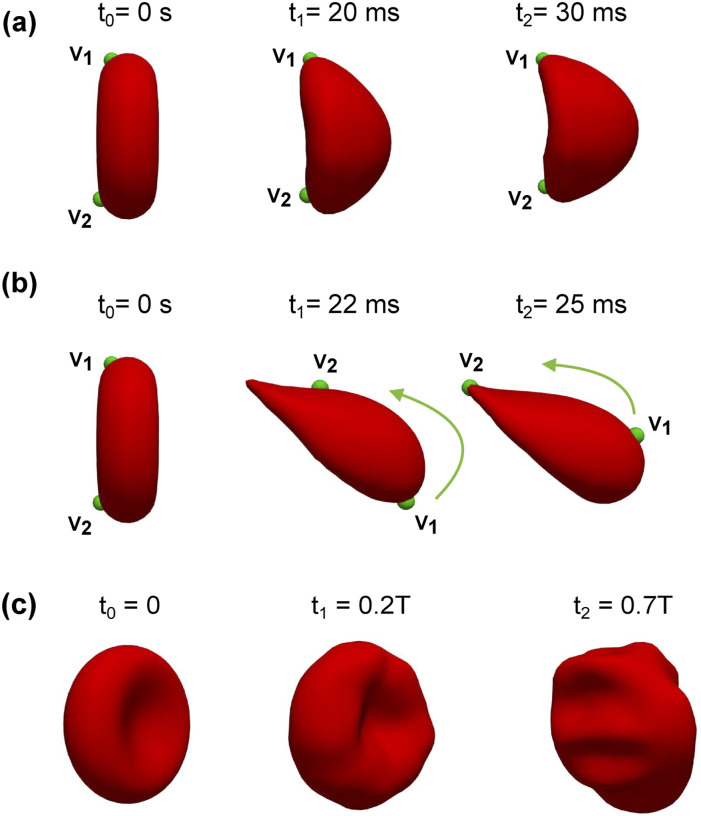
Transitions from the idealized shape to realistic shapes under the impact of a constant shear rate (*I*_0_) in (a) croissant shape (*I*_0_*U*_3_*r*_1_*χ*_1_) and (b) slipper shape (*I*_0_*U*_4_*r*_3_*χ*_1_). The RBC membrane only exhibits the tank-treading effect in the slipper shape (*I*_0_*U*_4_*r*_3_*χ*_1_), which is characterized by the motions of two Lagrangian markers *V*_1_ and *V*_2_. The slipper shape is maintained by the counterclockwise rotation (the green arrow) of the cellular membrane around the RBC’s centroid. The multilobe shape appears (c) under the oscillatory flow (*I*_*s*_*ψ*_6_*r*_1_*χ*_3_) during the backward phase (0.7*T*).

Under constant shear rate conditions (*I*_0_), as described in Sec. II G 1, two districts of the RBC shape are observed: (*i*) the croissant shape (*I*_0_*U*_3_*r*_1_*χ*_1_‐$\bar{\dot{\gamma_{0}}}=200\;s^{-1}$) and (*ii*) the slipper shape (*I*_0_*U*_4_*r*_3_*χ*_1_—$\bar{\dot{\gamma_{0}}}=600\;s^{-1}$), as shown in Fig. 7.

Under low shear rate (*I*_0_*U*_3_*r*_1_*χ*_1_), the RBC is initially placed along the centerline of the microchannel (discocyte shape). As the RBC interacts with the incoming flow, it deforms and eventually transitions to a croissant shape, as shown in Fig. 7(a). The terminal shape (croissant) is attained as the RBC continues to propagate along the channel’s symmetry axis. Note that the croissant shape, in this case, is not fully axis-symmetric. In addition, the croissant shape observed in this study is comparable with the parachute shape from our previous work,^29^ although not identical due to differences in channel geometry and flow conditions.

Under high shear rate (*I*_0_*U*_4_*r*_3_*χ*_1_), the RBC transitions to the slipper shape, as shown in Fig. 7(b), which exhibits a bistability mode with tank-treading behavior. Note that the RBC is placed at a radial shift *r*_3_ = 0.7 *µ*m. Thus, the initial location of the RBC is not at the channel’s symmetry axis. The tank-treading effect is a complex dynamic in which the RBC membrane propagates axially along the channel while it rotates around its own center of mass, as seen in Fig. 7(b). A counterclockwise rotation is observed as indicated by the locations of two membrane particles (Lagrangian points—*V*_1_ and *V*_2_) at different time instances (*t*_1_ = 22 ms and *t*_2_ = 25 ms).

In both the croissant or slipper shapes, the transition from the initial shape (discocyte) to the terminal shape (either croissant or slipper) occurs within around 30 ms, as seen in Fig. 7. Our simulation results agree well with the corresponding experimental data of Ref. 4 as well as those described in recent experiments on RBC transient dynamics.^52,53^ Furthermore, these transitions are in good agreement with the shape diagram^54^ for different capillary numbers and confinements. In conclusion, our simulations are able to replicate the dynamics of the croissant and slipper shapes excellently.

**FIG. 8.**
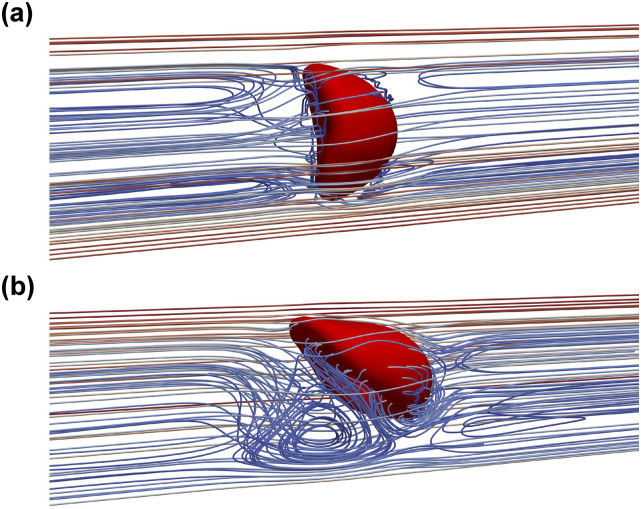
Extracellular flow patterns for (a) the croissant shape (*I*_0_*U*_3_*r*_1_*χ*_1_) and (b) the slipper shape (*I*_0_*U*_4_*r*_3_*χ*_1_). The flow streamlines are reconstructed using the co-moving frame method, as discussed in Sec. III B 1. The tank-treading effect induces a closed vortex to form on the upstream side of the RBC.

The extracellular patterns of the croissant and slipper shapes also agree well with the experimental data.^4,26^ The extracellular flow pattern can be visualized by reconstructing the relative flow velocity field^26^ (co-moving frame). The relative velocity is defined as the difference between the flow velocity and the RBC’s centroid velocity, as shown in Fig. 8. In the croissant shape (*I*_0_*U*_3_*r*_1_*χ*_1_), the velocity streamlines closely resemble an axi-symmetrical flow pattern [Fig. 8(a)]. The downstream side of the RBC membrane deforms significantly, whereas the upstream side barely changes, as depicted in Fig. 8(b). In the slipper shape (*I*_0_*U*_4_*r*_3_*χ*_1_), there exists an asymmetrical vortical structure in the vicinity of the RBC membrane. As the slipper shape emerges, a fully closed vortex ring is created by a separated flow region, which is close to the channel wall. In short, the emergence of the RBC shape dictates the extracellular flow pattern.

### 2. *Propulsion of RBC under stepwise* *oscillatory flows* *(I*_*s*_*)*

Under stepwise flow waveform (*I*_*s*_), our simulation results agree well with the propulsion step map (Δ*x*_*c*_, *Ca*^*f*^), which was developed^13^ for both channels *χ*_2_ = 0.5 and *χ*_3_ = 0.38. In both cases, the propulsion step (Δ*x*_*c*_) is observed to monotonically increase with the values of *Ca*^*f*^. However, Δ*x*_*c*_ is higher in the lower confinement channel (*χ*_3_), which indicates the importance of channel confinement.

In all simulation cases (*I*_*s*_*ψ*_1_*r*_1_*χ*_3_, *I*_*s*_*ψ*_2_*r*_1_*χ*_3_, *I*_*s*_*ψ*_3_*r*_1_*χ*_3_, *I*_*s*_*ψ*_4_*r*_1_*χ*_3_, *I*_*s*_*ψ*_5_*r*_1_*χ*_3_, and *I*_*s*_*ψ*_6_*r*_1_*χ*_3_), the RBC transitions from the idealized discocyte to the biconcave shape during the forward phase $\left(0<t<\frac{T}{5}\right)$ with all values of the peak forward flow (*ψ*_*f*_ = 1.05 mm s^−1^ to *ψ*_*f*_ = 4.34 mm s^−1^), as shown in Fig. 7(c). Strikingly, the complex multilobe shape emerges during the backward phase *T*_*b*_. The elastic response of the RBC membrane to the oscillatory flow during the cycle *T* is depicted for the case *I*_*s*_*ψ*_6_*r*_1_*χ*_3_ in Fig. 7(c). The reversal of the flow direction during *T*_*b*_ results in membrane buckling and stretching, which give rise to the multilobe shape. Note that these cases are initially placed at the channel center (*r* = *r*_1_ = 0). Therefore, it is possible to induce complex dynamics of RBCs in a confined channel by changing the inflow waveform only.

### C. The impact of sinusoidal flows on RBC dynamics

#### 1. The emergence of RBC shapes

**FIG. 9.**
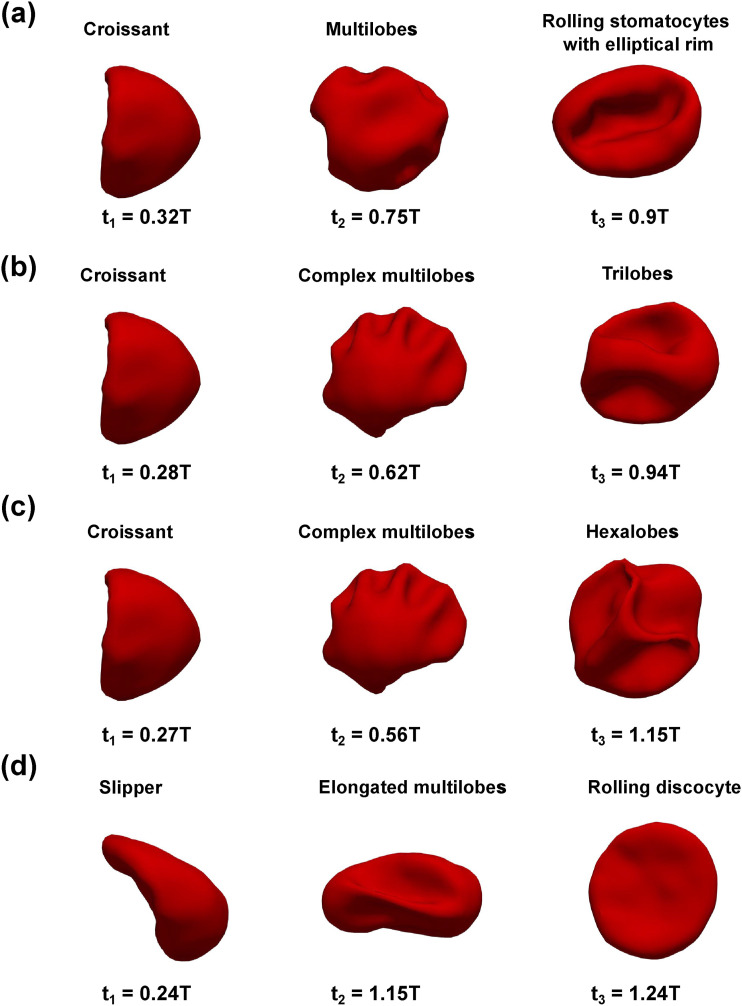
Emergence of complex shapes induced by different inlet sinusoidal waveforms at different time instances in the flow cycle *t*_1_ (end of forward phase), *t*_2_ (during the resting period), and *t*_3_ (backward flow phase). (a) *I*_1_*U*_1_*r*_1_*χ*_1_, (b) *I*_3_*U*_2_*r*_1_*χ*_1_, (c) *I*_4_*U*_2_*r*_1_*χ*_1_, and (d) *I*_4_*U*_3_*r*_3_*χ*_1_.

The sinusoidal flow waveform (*I*_1_, *I*_2_, *I*_3_, and *I*_4_) further adds complexity to the membrane dynamics as the shape of RBC is highly sensitive to the extracellular flow condition. As a result of the pulsatile flow condition, the RBC shape continuously responds to the applied flow in the channel. Our simulations show that the RBC alternates its shapes in one of the following types: (1) croissant; (2) slipper; (3) trilobes; (4) simple/complex/elongated multilobes; (5) rolling stomatocytes; (6) hexalobes; and (7) rolling discocyte, as shown in Fig. 9 and Tables VIII–XI. The emergence of each type will be discussed in the following.

**TABLE VIII.** Summary of the RBC morphology transition sequences recorded at different time instances $\frac{t}{T}$ under *I*_1_ waveform, and different flow velocities *U*_1_, *U*_2_, *U*_3_ and radial shift *r*_1_, *r*_2_, *r*_3_. Here, the numerical value in the table represents the first time instance that the RBC deformed shape appears. The acronyms *C*, *S*, *CM*, *M*, *T*, *RS*, *EM*, and *RD* represent the croissant, slipper, complex multilobes, multilobes, trilobes, rolling stomatocytes, elongated multilobes, and rolling discocyte, respectively. The exact numeric value of *U*_1_, *U*_2_, *U*_3_ and *r*_1_, *r*_2_, *r*_3_ are shown in Table VI.

	C	S		CM	M	T	RS	EM	RD		
Waveform (*I*_1_)	*r*_1_	*r*_2_	*r*_3_	*r*_1_	*r*_1_	*r*_1_	*r*_1_	*r*_1_	*r*_1_	*r*_2_	*r*_3_
*U*_1_	0.32	0.3	0.3	⋯	0.75	⋯	0.9	⋯	1.8	1.25	1.25
*U*_2_	0.21	0.2	0.2	0.5	⋯	0.8	⋯	1.2	⋯	1.21	1.21
*U*_3_	0.21	0.2	0.2	0.5	⋯	0.8	⋯	1.2	⋯	1.21	1.21

**TABLE IX.** Summary of the RBC morphology transition sequences recorded at different time instances $\frac{t}{T}$ under *I*_2_ waveform, and the different flow velocities *U*_1_, *U*_2_, *U*_3_ and initial placements *r*_1_, *r*_2_, *r*_3_. Here, the numerical value in the table represents the first time instance that the RBC deformed shape appears. The acronyms *C*, *S*, *CM*, *EM*, and *RD* represent the croissant, slipper, complex multilobes, elongated multilobes, and rolling discocyte, respectively. The exact numeric value of *U*_1_, *U*_2_, *U*_3_ and *r*_1_, *r*_2_, *r*_3_ are shown in Table VI.

Waveform (*I*_2_)	C	S		CM	EM		RD		
	*r*_1_	*r*_2_	*r*_3_	*r*_1_	*r*_2_	*r*_3_	*r*_1_	*r*_2_	*r*_3_
*U*_1_	0.27	0.29	0.29	0.9	⋯	⋯	1.55	1.33	1.33
*U*_2_	0.2	0.22	0.22	1.06	⋯	⋯	1.44	1.3	1.3
*U*_3_	0.2	0.22	0.22	1.06	1.16	1.16	1.8	1.3	1.3

**TABLE X.** Summary of the RBC morphology transition sequences recorded at different time instances $\frac{t}{T}$ under *I*_3_ waveform, and the different flow velocities *U*_1_, *U*_2_, *U*_3_ and initial placements *r*_1_, *r*_2_, *r*_3_. Here, the numerical value in the table represents the first time instance that the RBC deformed shape appears. The acronyms *C*, *S*, *CM*, *T*, *EM*, and *RD* represent the croissant, slipper, complex multilobes, trilobes, elongated multilobes, and rolling discocyte, respectively. The exact numeric value of *U*_1_, *U*_2_, *U*_3_ and *r*_1_, *r*_2_, *r*_3_ are shown in Table VI.

Waveform (*I*_3_)	C	S		CM	T	EM		RD		
	*r*_1_	*r*_2_	*r*_3_	*r*_1_	*r*_1_	*r*_2_	*r*_3_	*r*_1_	*r*_2_	*r*_3_
*U*_1_	0.34	0.27	0.27	0.58	⋯	⋯	⋯	1.35	1.35	1.35
*U*_2_	0.28	0.22	0.22	0.62	0.94	⋯	⋯	1.8	1.32	1.32
*U*_3_	0.28	0.22	0.22	0.62	⋯	1.18	1.18	1.4	1.32	1.32

**TABLE XI.** Summary of the RBC morphology transition sequences recorded at different time instances $\frac{t}{T}$ under *I*_4_ waveform, and the different flow velocities *U*_1_, *U*_2_, *U*_3_ and initial placements *r*_1_, *r*_2_, *r*_3_. Here, the numerical value in the table represents the first time instance that the RBC deformed shape appears. The acronyms *C*, *S*, *CM*, *EM*, *RD*, and *HX* represent the croissant, slipper, complex multilobes, elongated multilobes, rolling discocyte, and hexalobes, respectively. The exact numeric value of *U*_1_, *U*_2_, *U*_3_ and *r*_1_, *r*_2_, *r*_3_ are shown in Table VI.

Waveform (*I*_4_)	C	S		CM	EM		RD			HX
	*r*_1_	*r*_2_	*r*_3_	*r*_1_	*r*_2_	*r*_3_	*r*_1_	*r*_2_	*r*_3_	*r*_1_
*U*_1_	0.32	0.31	0.31	0.6	⋯	⋯	1.38	1.27	1.27	⋯
*U*_2_	0.27	0.24	0.24	0.56	⋯	⋯	1.5	1.24	1.24	1.15
*U*_3_	0.27	0.24	0.24	0.56	1.15	1.15	1.1	1.24	1.24	⋯

In all cases, the RBC evolves to the croissant (*C*) or the slipper (*S*) shape during the forward phase (0 < *t* < *T*_*f*_) of the flow cycle $\left(\frac{t}{T}\approx 0.25\right)$ , as shown in Tables VIII–XI. This process is demonstrated for the cases *I*_1_*U*_1_*r*_1_*χ*_1_, *I*_3_*U*_2_*r*_1_*χ*_1_, *I*_4_*U*_2_*r*_1_*χ*_1_, and *I*_4_*U*_3_*r*_3_*χ*_1_, as seen in Fig. 9 (first column). Note that the transition to *C* or *S* mode from the biconcave shape is dependent on the value of the radial shift (*r*). As shown in Tables VIII–XI, the *S* mode appears only when the RBC is initially placed not exactly at the cross-sectional center (*r* > 0) (for example, *I*_4_*U*_3_*r*_3_*χ*_1_). The RBC remains in *C* mode during the forward phase if it is initially placed at the cross section center (*r* = 0) regardless of the bulk flow waveform *I*_1_, *I*_2_, *I*_3_, or *I*_4_ as seen in cases *I*_1_*U*_1_*r*_1_*χ*_1_, *I*_3_*U*_2_*r*_1_*χ*_1_, and *I*_4_*U*_2_*r*_1_*χ*_1_. In brief, the croissant and the slipper shapes exist during the forward phase and their emergence depends on the radial shift (*r*).

The RBC transitions from the simple shapes (croissant and slipper) toward more complex shapes (trilobes, simple/complex/elongated multilobes, rolling stomatocytes, hexalobes, and rolling discocyte) later in the flow cycle during the resting/reverse periods $\left(\frac{t}{T}>0.5\right)$ , as shown in Tables VIII–XI and Fig. 9 (middle column). The shape transformation is initiated by the buckling of the RBC membrane, which takes place in the resting interval (*T*_*r*_) (see Fig. 5) of the flow. As a result of the change in flow direction in *T*_*b*_, the RBC experiences considerable stretching and compression, leading to significant alterations in its membrane shape, as seen in Fig. 9. Note that the RBC does not return to either *C* or *S* mode at the beginning of the second cycle *T* < *t* < 2*T*, as shown in Tables VIII–XI. Thus, the shape transition process is irreversible even if the inflow waveform is symmetric (*I*_1_), as shown in Table VIII.

#### 2. The impacts of the initial position (the radial shift—*r*)

Our results in Fig. 9 show that the initial position (*r*) plays a critical role in the emergence of RBC shapes. The RBC is placed initially at the channel axis *r* = *r*_1_ = 0 under both the symmetric (*I*_1_*U*_1_*r*_1_*χ*_1_) and asymmetric (*I*_3_*U*_2_*r*_1_*χ*_1_ and *I*_4_*U*_2_*r*_1_*χ*_1_) waveforms shown in Figs. 9(a)–9(c), respectively. In these cases (*I*_1_*U*_1_*r*_1_*χ*_1_, *I*_3_*U*_2_*r*_1_*χ*_1_, and *I*_4_*U*_2_*r*_1_*χ*_1_), the RBCs all transition sequentially from the croissant shape toward the multilobes/complex multilobes and finally one of the poly-lobes (trilobes, rolling stomatocytes, or hexalobes). When *r* = *r*_3_ > 0 in the case *I*_4_*U*_3_*r*_3_*χ*_1_, the RBC mostly remains in the slipper shape during the forward phase (*t* < *T*_*f*_), as seen in Fig. 9(d). It transitions toward the elongated multilobe during the backflow phase (*T*_*f*_ + *T*_*r*_ < *t* < *T*). Finally, the RBC becomes a rolling discocyte in the second cycle (*t* ≈ 1.2*T*). Note that *r*_3_ is a small value (0.7 *µ*m) and thus the shape transition process is strongly sensitive to the initial placement of the RBC. The dependence of RBC dynamics on the value of *r* is explained in the following.

**FIG. 10.**
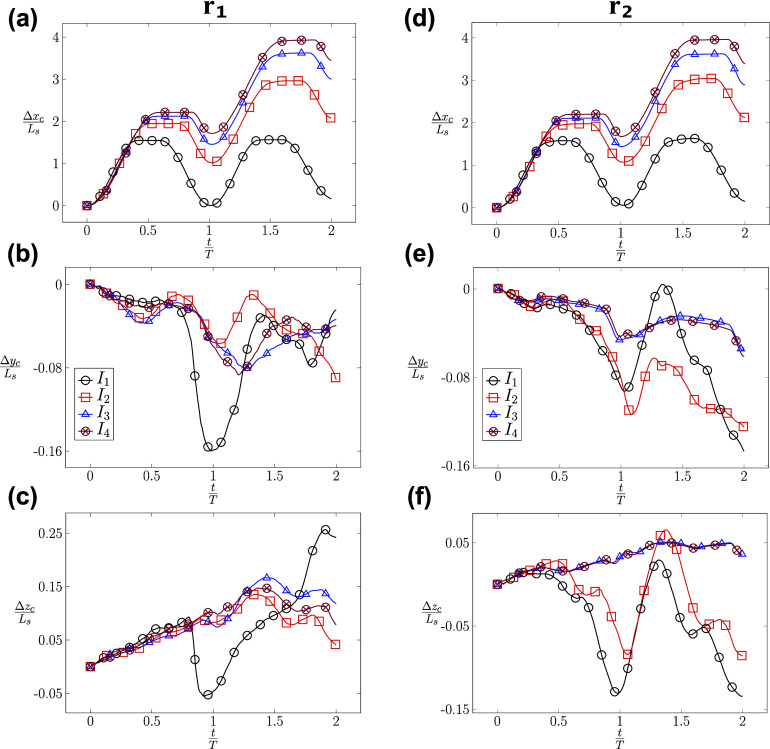
Impacts of the radial shift *r* on the time evolution of RBC’s centroid displacement (Δ*x*_*c*_, Δ*y*_*c*_, Δ*z*_*c*_), which are measured in units of the length scale *L*_*s*_. The evolution of the centroid position is examined under two conditions: (i) centered initial position *r* = *r*_1_ = 0 [left column—(a)–(c)] for the cases *I*_1_*U*_1_*r*_1_*χ*_1_, *I*_2_*U*_1_*r*_1_*χ*_1_, *I*_3_*U*_1_*r*_1_*χ*_1_, and *I*_4_*U*_1_*r*_1_*χ*_1_ and (ii) off-centered initial position *r* = *r*_2_ = 0.4 *µ*m [right column—(d)–(f)] for the cases *I*_1_*U*_2_*r*_2_*χ*_1_, *I*_2_*U*_2_*r*_2_*χ*_1_, *I*_3_*U*_2_*r*_2_*χ*_1_, and *I*_4_*U*_2_*r*_2_*χ*_1_, as described in Table VI.

When the RBC is positioned at the centerline (*r* = 0) of the channel, its migration is highly dependent on the type of inflow waveform. When the RBC is subjected to a symmetric waveform (*I*_1_) as shown in Fig. 10(a), it oscillates around its initial position with a minimal migration along the channel (axial) direction. However, as the inflow profile transitions to an asymmetric waveform (*I*_2_, *I*_3_, and *I*_4_) with an increasing *T*_*f*_, the RBC gains more momentum during the forward phase and propels far away from its initial position, as shown in Fig. 10(a) (left). The value of the axial displacement (Δ*x*_*c*_) (t) increases sequentially from *I*_1_, *I*_2_, *I*_3_ to *I*_4_. Δ*x*_*c*_ reaches a maximum value of ∼4 × *L*_*s*_ at the end of the second cycle for the case *I*_4_. In the lateral direction, Δ*y*_*c*_ reaches a value of ∼0.16 × *L*_*s*_ at the end of the first cycle for the symmetric waveform (*I*_1_), as shown in Fig. 10(b) (left). For the cases *I*_2_, *I*_3_, and *I*_4_, the values of Δ*y*_*c*_ remain comparably low $\left(\approx 0.08\times L_{s}\right)$ during the cycles. Furthermore, the vertical displacement Δ*z*_*c*_ seen in Fig. 10(c) follows a monotonically upward trend throughout the second cycle. This results in a maximum vertical displacement Δ*z*_*c*_ of ∼0.25 × *L*_*s*_ for *I*_1_. For other waveforms (*I*_2_, *I*_3_, and *I*_4_), a smaller upward trend is observed with a vertical displacement Δ*z*_*c*_ ≈ 0.08 × *L*_*s*_ at the end of the first cycle. During the second cycle, the cell is observed to migrate downward. In summary, the symmetric waveform (*I*_1_) leads to a maximum displacement in the transverse directions while the asymmetric waveforms (*I*_2_, *I*_3_, and *I*_4_) result in the maximum axial displacement for *r* = 0.

As the RBC is placed at the off-centered location (*r* = *r*_2_ = 0.4 *µ*m), as seen in Fig. 10(d), its axial migration is similar to ones with *r* = 0 [Fig. 10(a)]. The axial displacement (Δ*x*_*c*_) is dependent on the applied inflow waveform and also reaches 4 × *L*_*s*_ for the *I*_4_ waveform. These values indicate that the radial shift (*r*) does not significantly affect the axial migration of the RBC. However, the lateral migration of RBC in Figs. 10(e) and 10(f) does show a dependence of the applied waveform. The values of Δ*y*_*c*_ and Δ*z*_*c*_ reach 0.14 × *L*_*s*_ for *I*_1_ and *I*_2_ at the end of the second cycle, respectively. Surprisingly, the impact of the applied waveform seems to diminish as the value of *T*_*f*_ increases. Comparing the case *I*_3_*U*_2_*r*_2_*χ*_1_ and *I*_4_*U*_2_*r*_2_*χ*_1_ (*I*_3_ and *I*_4_), the values of Δ*y*_*c*_ and Δ*z*_*c*_ shown in Figs. 10(e) and 10(f) are nearly identical, resulting in a vertical displacement of ∼0.04 × *L*_*s*_. To summarize, the impact of the applied waveform is significant on the axial displacement of the RBC but it is milder on the lateral displacements when *r* > 0. The impact of the applied waveform diminishes as the forward time *T*_*f*_ increases.

#### 3. The impacts of the waveform (*I*)

**FIG. 11.**
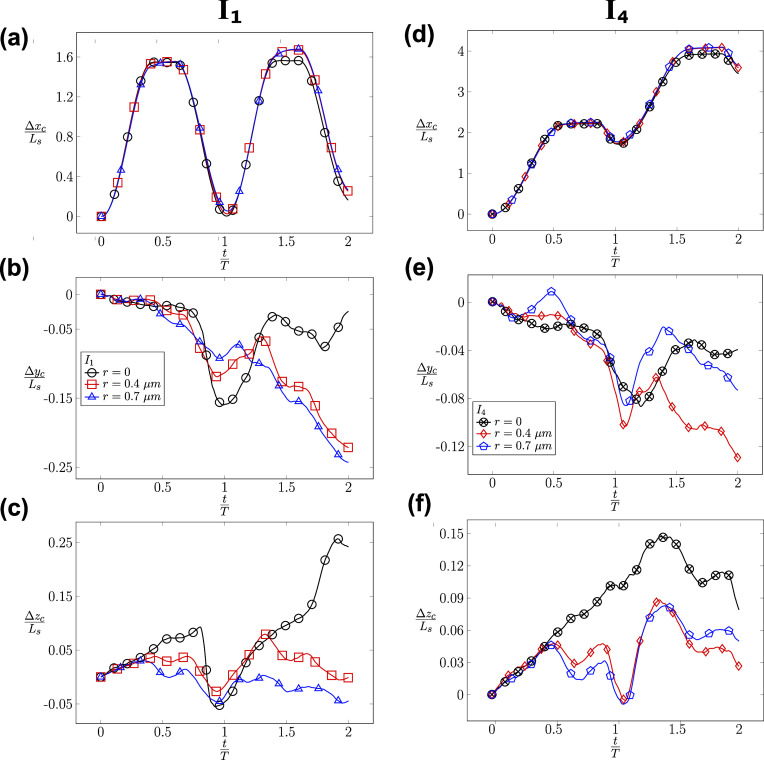
Impacts of the waveform (*I*) on the time evolution of RBC’s centroid displacement (Δ*x*_*c*_, Δ*y*_*c*_, Δ*z*_*c*_), which are measured in units of the length scale *L*_*s*_ under two conditions: (i) the symmetric *I*_1_ [left column—(a)–(c)] and (ii) the asymmetric *I*_4_ [right column—(d)–(f)] waveforms at different values of the radial shift *r*_1_, *r*_2_, and *r*_3_. The symmetric flow cases (left column) include *I*_1_*U*_1_*r*_1_*χ*_1_, *I*_1_*U*_1_*r*_2_*χ*_1_, and *I*_1_*U*_1_*r*_3_*χ*_1_. The asymmetric flow cases (right column) include *I*_4_*U*_1_*r*_1_*χ*_1_, *I*_4_*U*_1_*r*_2_*χ*_1_, and *I*_4_*U*_1_*r*_3_*χ*_1_ cases. The exact values of *r*_1_, *r*_2_, and *r*_3_ are described in Table VI.

It is striking to observe the irreversible dynamics of RBC in Fig. 11. When the RBC is subjected to the symmetric waveform (*I*_1_) with different radial shifts *r* = *r*_1_, *r*_2_, and *r*_3_, the RBC oscillates around its initial position with a minimal displacement along the axial direction as shown in the cases *I*_1_*U*_1_*r*_1_*χ*_1_, *I*_1_*U*_1_*r*_2_*χ*_1_, and *I*_1_*U*_1_*r*_3_*χ*_1_. Despite the inflow waveform being completely symmetrical (a sine function—*I*_1_), the axial position of the RBC in Fig. 11(a) (left column) shows a net positive value of the displacement Δ*x*_*c*_ at the end of the first (*t* = *T*) and second cycle (*t* = 2*T*) even when there is no radial shift (*r* = 0). Although small, this positive value of Δ*x*_*c*_ indicates that the RBC does not go back exactly to its initial location, which is Δ*x*_*c*_(*t* = 0) = 0. At all values of the radial shift of *r* = 0, 0.4, and 0.7 *µ*m, this irreversible dynamics is even more evident, as shown in the lateral displacements in Figs. 11(b) and 11(c). The magnitudes of Δ*y*_*c*_ and Δ*z*_*c*_ are comparable for all values of *r* during the cycles. For the case *I*_1_*U*_1_*r*_1_*χ*_1_ (*r* = 0), the value of Δ*y*_*c*_ reaches a value of ∼0.16 × *L*_*s*_ at the end of the first cycle. For the cases *I*_1_*U*_1_*r*_2_*χ*_1_ and *I*_1_*U*_1_*r*_3_*χ*_1_, the values of Δ*y*_*c*_ and Δ*z*_*c*_ reach ∼0.25 × *L*_*s*_ at the end of the second cycle. In the vertical direction (*z*_*c*_) shown in Fig. 11(c), the well-centered RBC (*r* = 0) is influenced by the change of flow direction, which is depicted by the upward and downward trends in the first cycle. However, the cell follows a dominant upward trend during the entire second cycle, resulting in a lateral migration of around 0.25 × *L*_*s*_. Therefore, there exists a significant lateral migration of the RBC during its propagation regardless of its initial position under the symmetrical waveform condition (*I*_1_). In conclusion, a symmetrical flow waveform (*I*_1_) results in minimal propulsion along the axial direction but a significant lateral migration.

Under asymmetric waveform *I*_4_, the RBC propels along the channel direction with an axial displacement of ∼2 × *L*_*s*_ in each cycle, as shown in Fig. 11(d). As the waveform becomes asymmetric with a longer forward phase, the RBC does not go back significantly during the reverse phase. It rather remains at a displacement value of Δ*x*_*c*_ ≈ 1.9 × *L*_*s*_ at the end of the first cycle. It continues to propel in the second cycle up to Δ*x*_*c*_ ≈ 4.0 × *L*_*s*_. Surprisingly, the lateral displacements of the RBC (Δ*y*_*c*_, Δ*z*_*c*_) are smaller in comparison with ones in the symmetric case (*I*_1_). The values of (Δ*y*_*c*_, Δ*z*_*c*_) are within 0.15 × *L*_*s*_ for all cases *I*_4_*U*_1_*r*_1_*χ*_1_, *I*_4_*U*_1_*r*_2_*χ*_1_, and *I*_4_*U*_1_*r*_3_*χ*_1_, as shown in Figs. 11(e) and 11(f). In brief, the RBC propels significantly under the impact of the asymmetrical flow waveform *I*_4_ along the axial direction but it does not migrate significantly in the lateral directions.

#### 4. Extracellular flow dynamics at the vicinity of the RBC under oscillatory flows

**FIG. 12.**
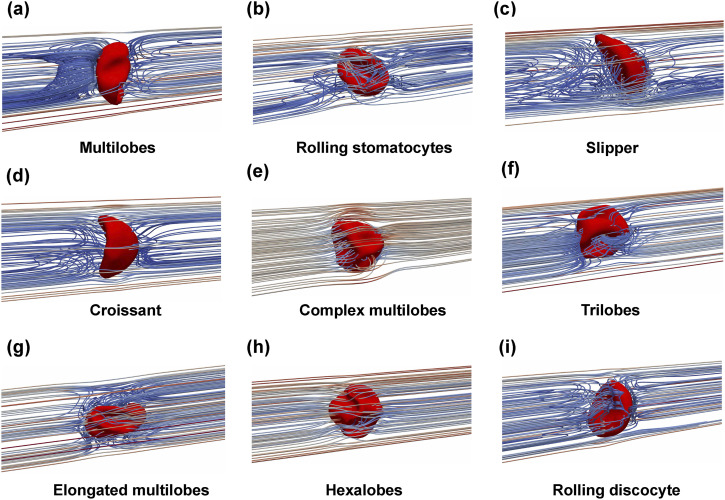
Flow patterns surrounding the RBC under different membrane shapes (also see Fig. 9). The flow patterns are visualized using streamlines of the velocity field in the co-moving frame with the RBC (also see Sec. III B 1). The representative flow patterns are shown for (a) the multilobes (*I*_1_*U*_1_*r*_1_*χ*_1_); (b) rolling stomatocytes (*I*_1_*U*_1_*r*_1_*χ*_1_); (c) slipper (*I*_1_*U*_3_*r*_3_*χ*_1_); and (d) croissant (*I*_1_*U*_3_*r*_1_*χ*_1_); (e) complex multilobes (*I*_1_*U*_3_*r*_1_*χ*_1_); (f) trilobes (*I*_1_*U*_3_*r*_1_*χ*_1_); (g) elongated multilobes (*I*_1_*U*_3_*r*_1_*χ*_1_); (h) hexalobes (*I*_4_*U*_2_*r*_1_*χ*_1_); and (i) rolling discocyte (*I*_1_*U*_1_*r*_2_*χ*_1_).

The emergence of the RBC shape has a close relationship with the flow pattern of the surrounding fluid (extracellular flow). Under the impact of the channel confinement, the deformation of RBC is well regulated by the flow waveform, which results in distinct extracellular flow patterns surrounding the RBC, as shown in Figs. 9 and 12. To highlight the impact of the RBC motion, the flow pattern is visualized in the co-moving frame with the RBC’s centroid (see Sec. III B 1). Thus, the flow streamlines are represented from the perspective of the RBC.

The case *I*_1_*U*_1_*r*_1_*χ*_1_ is selected to illustrate the evolution of flow pattern as the RBC deforms from a relatively simple shape to a more complicated shape, as depicted in Figs. 12(a) and 12(b). This case is chosen because the temporal variation of the waveform is completely symmetrical (*I*_1_). Moreover, the RBC is placed initially at the channel axis (*r* = *r*_1_ = 0) with the lowest forward velocity *ψ*_*f*_ = *U*_1_ = 1 mm s^−1^. In the case *I*_1_*U*_1_*r*_1_*χ*_1_, Fig. 12(a) revealed that the RBC has a multilobes shape at the end of the forward phase. The presence of the large lobes results in a more convoluted streamlines pattern during the resting phase. As the RBC undergoes a morphological transition to rolling stomatocytes at the end of the first cycle (*t* = 0.9*T*), the streamlines exhibit changes [Fig. 12(b)]. During the second cycle, the RBC gradually transforms into a rolling discocyte by the end of the second cycle (*t* = 1.8*T*).

The impact of the radial shift (*r*) on the RBC shape and the resulting flow pattern is significant. To highlight the impact of the initial location, the case *I*_1_*U*_3_*r*_3_*χ*_1_ was selected to visualize the flow patterns. As shown in Fig. 12(c), due to the off-centered initial location (*r* > 0), the slipper shape emerges during the forward phase. A closed vortex ring is also observed downstream of the RBC as the flow velocity reaches its maximum magnitude in the forward phase. This phenomenon is similar to the one observed in the constant shear rate case (*I*_0_*U*_4_*r*_3_*χ*_1_ with *U*_4_ = 6 mm s^−1^) in Fig. 8(b). This is remarkable since the peak flow *ψ*_*f*_ is rather three times lower in this case *ψ*_*f*_ = *U*_3_ = 2 mm/s.

The case *I*_1_*U*_3_*r*_1_*χ*_1_ [Figs. 12(d)–12(g)] is selected to illustrate further the impact of the peak forward flow *ψ*_*f*_. In this case, the peak velocity is *ψ*_*f*_ = *U*_3_ = 2 mm s^−1^. In comparison with the case *I*_1_*U*_1_*r*_1_*χ*_1_ [Figs. 12(a) and 12(b)], only the value of the peak velocity *ψ*_*f*_ is increased. However, the RBC shape evolves in a completely different sequence, as opposed to the case *I*_1_*U*_1_*r*_1_*χ*_1_. During the forward phase, the RBC transitions quickly to the croissant shape in Fig. 12(d) at (*t* = 0.28*T*), just after the peak forward flow. The flow patterns are similar to those observed under case *I*_0_*U*_3_*r*_1_*χ*_1_ in Fig. 8(a). During the resting period (*T*_*f*_ < *T* < *T*_*f*_ + *T*_*r*_), the flow velocity surrounding the cell decreased notably and the complex multilobes shape emerges, as seen in Fig. 12(e). The flow pattern is perturbed minimally surrounding the RBC as its shape turns to the trilobes shape, as depicted in Fig. 12(f). During the forward flow phase of the second cycle, the elongated multilobes appear (*t* = 1.2*T*).

The overall dynamics of the RBC over the cycles depend significantly on the radial shift. As demonstrated in Tables VIII–XI, the multilobes shape appears at the beginning of each cycle if the RBC is located initially at the channel center *r* = 0. Under the specific condition of the case *I*_4_*U*_2_*r*_1_*χ*_1_, the multilobes shape can further transform into the hexalobes shape during the forward flow phase of the second cycle (*t* = 1.15*T*), as shown in Table XI. Its corresponding flow patterns are shown in Fig. 12(h), in which the extracellular flow was observed to exhibit a minimal disturbance around the hexalobes shape as the RBC. For the majority of the off-centered cases (*r* > 0), the rolling discocyte case is found the most commonly, as shown in Tables VIII–XI. The flow pattern around a discocyte is exemplified in Fig. 12(i) for the case *I*_1_*U*_1_*r*_2_*χ*_1_ at *t* = 1.25*T*. Here, the flow streamlines show a distinct separation of upstream and downstream regions.

## IV. DISCUSSION

Due to the membrane flexibility, RBC deforms swiftly under the shear flow.^11^ This morphological feature can be exploited to understand the mechanical properties of the RBC membrane^53^ and thus it has the potential to identify the pathological changes^52^ of RBC’s membrane. However, the exact mechanism of this response is not yet fully understood. In addition, experiments^14^ have shown a strong proof-of-principle in which unsteady shear flows characterized by forward–backward profiles were exploited to produce distinct deformation-induced actuation of cells with different elastic properties. This is significant and is potentially useful in cell sorting and separation applications. In this work, we explore the impacts of the unsteady shear rate to control cell deformation and migration in microchannels.

Our numerical method is based on the continuum–particle coupling,^29^ which allows the simulations of RBC dynamics under physiological conditions. Our numerical results show excellent agreements with available *in vitro* and computational data both in cellular mechanics and extracellular flow pattern of the blood plasma.^4,26,51^ While most previous studies^30,31^ have only focused on the impact of constant shear rate on the dynamics of the RBCs, our results show that the unsteady shear rate can induce complex RBC’s morphology in confined channels, as discussed in the following.

### A. The emergence of the croissant shape and the slipper shape under a constant shear rate $\bar{\dot{\gamma_{0}}}$)

Recent studies^4,26^ in rectangular microchannels, which are identical to our channels as shown in Fig. 3 and Table III, suggest that the emergence of the RBC shape is dependent on the radial shift (*r*—see Fig. 3 for its definition). In previous studies,^4,26^ the croissant shape emerged at $\bar{\dot{\gamma_{0}}}<300\;s^{-1}$ if the RBC is placed exactly at the channel’s center (*r* = 0). On the other hand, the slipper shape emerged whenever the RBC was not placed exactly at the centerline (*r* > 0). The RBC was found to exhibit a (tank-treading) slipper shape at sufficiently high shear rate $\left(\bar{\dot{\gamma_{0}}}\approx 500\;s^{-1}\right)$ and off-centered placement (*r* > 0).^4,26^ In cylindrical microchannels,^17^ similar observations were confirmed, albeit at lower shear rates $\left(0<\bar{\dot{\gamma_{0}}}<80\;s^{-1}\right)$. These studies point to the importance of the radial shift in regulating RBC dynamics. Our results shown in Fig. 7 agree with these observations (also see Tables III and IV). Our results show the appearance of croissant-to-slipper transition as the capillary number (and thus $\bar{\dot{\gamma_{0}}}$) increases from 0.1 to 0.37 for a confinement of *χ* = 0.65. The croissant shape emerges when the initial position of the RBC is placed exactly at the channel centerline at a sufficiently low shear rate (*Ca* = 0.1). When the shear rate is increased to *Ca* = 0.37, the slipper shape emerges. Furthermore, our model is able to capture the intricate dynamics of the tank-treading motion, which is characterized by the rotation of the membrane at the shear rate of 600 s^−1^, as illustrated in Fig. 7. Therefore, our results further confirm the importance of the radial shift in the croissant-to-slipper transition.

### B. The impact of time-varying shear rate $\bar{\dot{\gamma \left(t\right)}}$ on RBC shape

When the inflow varies in a stepwise manner, as seen in Fig. 4, the shear rate $\left(\bar{\dot{\gamma }}\right)$ changes as a function of time $\bar{\dot{\gamma }}\left(t\right)$ with distinct forward (*T*_*f*_) and backward (*T*_*b*_) time phases. In all cases (*I*_*s*_*ψ*_1_*r*_1_*χ*_2_, *I*_*s*_*ψ*_2_*r*_1_*χ*_2_, *I*_*s*_*ψ*_3_*r*_1_*χ*_2_, *I*_*s*_*ψ*_4_*r*_1_*χ*_3_, *I*_*s*_*ψ*_5_*r*_1_*χ*_3_, and *I*_*s*_*ψ*_6_*r*_1_*χ*_3_), the RBC is placed exactly at the channel axis (*r* = *r*_1_ = 0). The shape transitions are accomplished through consistent transient stretching and compression of the membrane. This occurs as the RBC experiences forward and backward flow phases during the flow cycles. The RBC transitions from a discocyte shape toward a croissant shape during its forward propulsion, as shown in Fig. 7. Although the backward phase induces the buckling of the cellular membrane, the RBC shape remains relatively symmetrical with respect to the channel axis (multilobes) as shown in Fig. 7 at the end of *T*_*b*_. This is remarkable given that the maximum shear rate during the backward phase can be sufficiently large $\left({\bar{\dot{\gamma }}}_{f}=200\;s^{-1}\right)$ but this symmetry is still maintained. Comparing the case *I*_0_*U*_3_*r*_1_*χ*_1_ and *I*_0_*U*_4_*r*_3_*χ*_1_ in Table IV, our results suggest that the complete break of symmetry^52^ (slipper shape) is observed only when the radial shift exists (*r* > 0).

When applying different sinusoidal waveforms (*I*_1_, *I*_2_, *I*_3_, and *I*_4_) shown in Fig. 5, our results show the ubiquitous presence of croissant and slipper shapes across all shear rates (${\bar{\dot{\gamma }}}_{f}=100,150$, and 200 *s*^−1^). The slipper shape appears at (*t* ≈ 0.3*T*) whenever the RBC is placed off the channel’s axis (*r* > 0), as shown in Tables VIII–XI. Note that these waveforms are different in terms of the forward (*T*_*f*_) and backward (*T*_*b*_) phases, with the backward phase being the shortest in *I*_4_. As shown in Table VIII, the slipper shape is observed even when the waveform is completely symmetric (*I*_1_) given that *r* > 0, as in (a) *I*_1_*U*_1_*r*_2_*χ*_1_, (b) *I*_1_*U*_1_*r*_3_*χ*_1_, (c) *I*_1_*U*_2_*r*_2_*χ*_1_, (d) *I*_1_*U*_2_*r*_3_*χ*_1_, (e) *I*_1_*U*_3_*r*_2_*χ*_1_, and (f) *I*_1_*U*_3_*r*_3_*χ*_1_. Hence, our results indicate that the flow waveform does not affect the emergence of the RBC shape during the forward phase. Instead, the radial shift plays an essential role in this process.

It has been demonstrated^11^ that the presence of the discocyte shape is correlated with weak shear rates $\bar{\gamma_{0}}$. Under ${\bar{\dot{\gamma }}}_{0}<10\;s^{-1}$, the RBC typically maintains its discocyte shape with an 80% probability.^11^ However, as the shear rate gradually rises $\left(10\;s^{-1}\leq {\bar{\dot{\gamma }}}_{0}\leq 400\;s^{-1}\right)$, the likelihood of a discocyte shape decreases to 30%. This observation has been validated even when different viscosity ratios (*λ*)^12^ are considered. In our work, the discocyte shape is ubiquitously observed during the second flow cycle across all applied waveforms (*I*_1_, *I*_2_, *I*_3_, and *I*_4_) and shear rates (${\bar{\dot{\gamma }}}_{f}=100$, 150, and 200 s^−1^) with (*r* > 0) as shown in Tables VIII–XI. Therefore, our results further confirm that discocyte is the most common shape with the range shear rate less than 200 *s*^−1^.

In our study, complex shapes evolve from simpler shapes under the influence of time-dependent waveforms. In the experimental work^11^ under constant shear rates, stomatocytes shape was observed to dominate the RBC population (65%) when the shear rate was between $10\;s^{-1}<\bar{\dot{\gamma_{0}}}<400\;s^{-1}$. In our study, the elliptical-rim-shaped stomatocytes only emerge under symmetric waveform (*I*_1_) in the case *I*_1_*U*_1_*r*_1_*χ*_1_ under the peak shear rate of ${\bar{\dot{\gamma }}}_{f}=100\;s^{-1}$. At high constant shear rates $400\;s^{-1}<\bar{\dot{\gamma_{0}}}<2000\;s^{-1}$, experimental data^11^ showed that polylobes shape could emerge. This polylobes shape is characterized by a large number of lobes on the RBC surface, known as multilobes, trilobes, and hexalobes. In the current study, polylobes are also observed across all applied waveforms, given that the cell is placed initially at the channel axis (*r* = *r*_1_ = 0) even at weak shear rates $\left({\bar{\dot{\gamma }}}_{f}\leq 200\;s^{-1}\right)$ as in Fig. 9 and Tables VIII–XI. For example, the multilobes are observed with all waveforms. The trilobes are both observed in the symmetric waveform (*I*_1_*U*_2_*r*_1_*χ*_1_ and *I*_1_*U*_3_*r*_1_*χ*_1_—Table VIII) or the asymmetric waveform (*I*_3_*U*_2_*r*_1_*χ*_1_—Table X). The hexalobes shape only appears under the most asymmetric waveform with $\left(I_{4}U_{2}r_{1}\chi_{1}\right)-\bar{\dot{\gamma_{f}}}=150\;s^{-1}$, as shown in Table XI. In the previous work,^55^ the RBC shape has been reported to deform further into elongated shapes as the shear rate increases. Our results support this observation shown in Fig. 12(g) as the elongated multilobes appear during the backward flow phase. Examining Tables VIII–XI, our results suggest that this elongated shape is generally present regardless of the applied waveform but it only manifests under higher shear rates of at least ${\bar{\dot{\gamma }}}_{f}=150\;s^{-1}$. In particular, the elongated multilobes are observed under *I*_1_*U*_2_*r*_1_*χ*_1_, *I*_1_*U*_3_*r*_1_*χ*_1_, *I*_2_*U*_3_*r*_2_*χ*_1_, *I*_2_*U*_3_*r*_3_*χ*_1_, *I*_3_*U*_3_*r*_2_*χ*_1_, and *I*_3_*U*_3_*r*_3_*χ*_1_. In essence, our results strongly suggest that the complex shape can appear in a micro-channel even at weak shear rates given that the inflow is time-dependent.

While the radial shift is important for the emergence of the RBC shape, it is less important in controlling the cell displacement (the center of mass), as shown in Fig. 11. The background flow velocities vary largely over the cycle in the forward and reverse phases, depending on the flow waveform, as shown in Fig. 5. This variation dominates the plasma flow surrounding the RBC. Therefore, small radial shifts (0–0.7 *µ*m) play a minimal role in regulating the overall displacement of the RBC’s center of mass.

### C. Controlling lateral migration of cells with oscillatory flows

Microfluidic devices are typically used to isolate and separate cells.^56^ While these devices are promising for many cell-sorting applications,^57,58^ the main challenge is the difficulty in obtaining high-throughputs due to the required length of the microfluidic channels. Changing the geometrical design of channels^59^ has been proposed as one efficient way. Recent studies have shown that varying the shear rates in time^13,14^ can reduce the required length based on the concept of velocity lift,^60^ which is the factor that drives the RBC’s migration toward the center of the channel.

As the inertial effect is negligible at a very low Reynolds number (*R*_*e*_ ≈ 0.01), the flow is reversible for a rigid body. Thus, a rigid body will return to its initial position if the inflow conditions in the backward phase are reversed in the exact opposite way of its own during the forward phase. However, the RBC is not a rigid body and its membrane is highly flexible. Experiments^14^ have shown that the responses of RBCs are different from the ones of stiff beads in oscillatory flows. The RBC migration is observed to have a net actuation in oscillating flows whereas a stiff bead does not.

Our results in Figs. 10 and 11 for the symmetric waveform (*I*_1_) show that the RBC does not go back to its initial position at the end of the cycle. There is an axial shift of the RBC from its original position (Δ*x*_*c*_ ≠ 0) at the end of the cycle. Moreover, the RBC migrates significantly in the lateral cross section (Δ*y*_*c*_ ≫ 0 and (Δ*z*_*c*_ ≫ 0), as shown in Fig. 10. Our results in Figs. 11(b) and 11(e) indicate that the RBC undergoes lateral migration. This migration is particularly evident in the cases *I*_1_*U*_1_*r*_3_*χ*_1_ and *I*_4_*U*_1_*r*_3_*χ*_1_, where the cell migrates in the lateral direction for a distance of ∼0.25*L*_*s*_ and 0.13*L*_*s*_, respectively. Comparing the shapes of the waveform *I*_1_, *I*_2_, *I*_3_, and *I*_4_ in Fig. 5, our results suggest that significant lateral migration can be induced by adjusting the forward time interval (*T*_*f*_). With our sinusoidal waveforms shown in Fig. 5, *T*_*f*_ should be less than three times the backward flow phase *T*_*b*_ ($\frac{T_{f}}{T_{b}}<3$) to be able to induce significant lateral migration. This lateral migration in oscillatory flows might be used to separate cells selectively based on their mechanical properties.

Our findings in Figs. 8 and 12 show that the extracellular flow patterns are directly influenced by the dynamics of the RBC. Under a stationary condition in Fig. 8, the extracellular flow dynamics in the croissant shape are distinctively different from the ones of the slipper shape. In particular, the flow around the steady croissant shape was similar to that of a rigid sphere,^61^ in which the flow streamlines move nearly symmetrically inward and outward from the cell in the upstream and downstream sides, respectively. In contrast, a fully closed vortex ring (“bolus”) was observed downstream the cell for the slipper shape.^4^ Our results in Fig. 12 suggest that the extracellular flow patterns are far more complicated and highly dependent on the type of inflow waveform in time-dependent shear rates. It is important to note that the extracellular flow has been found to play an important role in drug delivery strategies^26^ due to its potential use of particle trapping. Therefore, our results suggest that controlling the inflow waveform either by adjusting the peak flow *ψ*_*f*_ or the shape of the waveform (specifically *T*_*f*_) might lead to the desired effects in delivering small particles (e.g., therapeutic nano-particles) to the RBCs.

### D. Limitations

While the current model has successfully simulated various RBC morphologies under different flow conditions, it is crucial to acknowledge its limitations. First, the current model assumes that the impact of the collision force of the cytosol particles [Eq. (8)] is negligible in determining the shape of the cellular membrane, as shown in many validations of Sec. III A with high shear rates. However, it is important to recognize that this collision force might have impacts on the dynamics of the membrane and cytosol under very low shear rates. Therefore, future work will need to develop new algorithms for the collision scheme.^30,40^ Second, analyzing the distribution of cytosol fluid particles and their conformational temperature could offer valuable insights into cytosol dynamics. In this work, the number of cytosol particles (*N*_*f*_) is set to be 100, which is much less than the number of membrane particles (*N*_*v*_). The coarse resolution of the cytosol fluid may lead to significant under-representation of hydrodynamic interactions and spatial gradients in the cytoplasm. Therefore, the intracellular flow cannot be reliably reproduced. In future studies, a larger number of *N*_*f*_ should be implemented to capture the intracellular flow patterns accurately. Third, previous work^62^ has shown that Eq. (A11) does not satisfy angular momentum conservation. This violation might induce inaccurate behaviors in simulating the dynamics of fluids such as the cytosol fluid. This issue needs to be investigated in future work. Fourth, the intracellular pressure coefficient *p*_*bs*_ = 4.12 Pa is chosen empirically, which is based on the range of RBC intracellular pressure.^63^ Further investigations might be needed to identify the optimal value of *p*_*bs*_. In addition, clustering of red blood cells is an important phenomenon, which determines the overall rheology of blood flow. Clusters of RBCs are formed by the adherence of individual cells, which have been shown to be dependent not only on the characteristics of the RBC membrane but also on the hydrodynamic condition of the plasma solution.^64^ While this clustering process is beyond the scope of this work, our methodology can be extended to study this phenomenon by incorporating the interaction forces between adjacent cell membranes. This issue will be addressed in our future work. Finally, our coupling methodology utilizes the sharp-interface immersed boundary method,^45^ which requires only the velocities on the cellular membrane as boundary conditions. In this work, the physical time step is chosen in the order of microseconds. Therefore, the thermal fluctuations of the velocity fields on the cellular membrane are fully resolved via the implicit momentum solver of the fractional step method (Sec. II E). Future studies need to be carried out to accelerate the convergence of the fractional step method for the Navier–Stokes equations and allow larger physical time steps.

## V. CONCLUSION

Transient dynamics of red blood cells (RBCs) in confined channels under oscillatory flows are investigated using our continuum–particle approach.^29^ To the best of our knowledge, it is the first time a hybrid continuum–particle approach is applied to simulate RBC dynamics under time-dependent shear rates. Our results reveal that the dynamics of RBCs are complex with different shaped modes that are beyond the usually observed croissant and slipper modes. Our results indicate that the extracellular flow pattern around the RBC is dependent on the RBC shape. Our results suggest that the oscillatory flow can be used to control and manipulate the dynamics of RBC by adapting the appropriate flow waveform. Our specific conclusions are given in the following.

• The RBC can transform into a variety of shapes, such as multilobes, trilobes, and hexalobes, by varying the sinusoidal waveform even when it is subjected to a relatively weak shear rate $\left(\bar{\dot{\gamma_{f}}}\leq 200\;s^{-1}\right)$.
• Simple shapes such as croissant, slipper, and rolling discocyte appear when the RBC is subjected to all waveforms. However, complex shapes such as rolling stomatocytes, trilobes, and hexalobes appeared only under specific conditions. In our study, the RBC transitions into eight shapes under the symmetric waveform (*I*_1_) and into five shapes under the asymmetric waveform (*I*_2_). Therefore, it is possible to attain a certain shape using an appropriate waveform.
• Under the symmetric flow waveform, the axial displacement of the RBC is rather minimal. However, the lateral displacements are significantly large. Under the asymmetric flow waveform, the RBC experiences a large axial displacement but small lateral displacements.
• The maximum lateral displacement of the RBC during its propagation depends on the initial radial shift (*r*). This maximum value is also dependent on the asymmetry of the flow waveform (*I*).
• The extracellular flow surrounding the RBC depends on its morphological shape. The flow pattern is thus distinct and unique for each shape.

In conclusion, the main contribution of our work is the demonstration for the potential control of RBC dynamics and the associated extracellular flow patterns in microfluidic channels via oscillatory flows. Our work can provide guidance for experimental design in future studies to investigate the RBC dynamics under time-dependent shear rates. In future work, we aim to leverage the capabilities of our novel hybrid continuum–particle framework to conduct detailed computational investigations of cellular-scale blood flow, complex microvascular networks,^65,66^ and pathological conditions such as sickle cell disease.^67^

## Figures and Tables

**FIG. 13.**
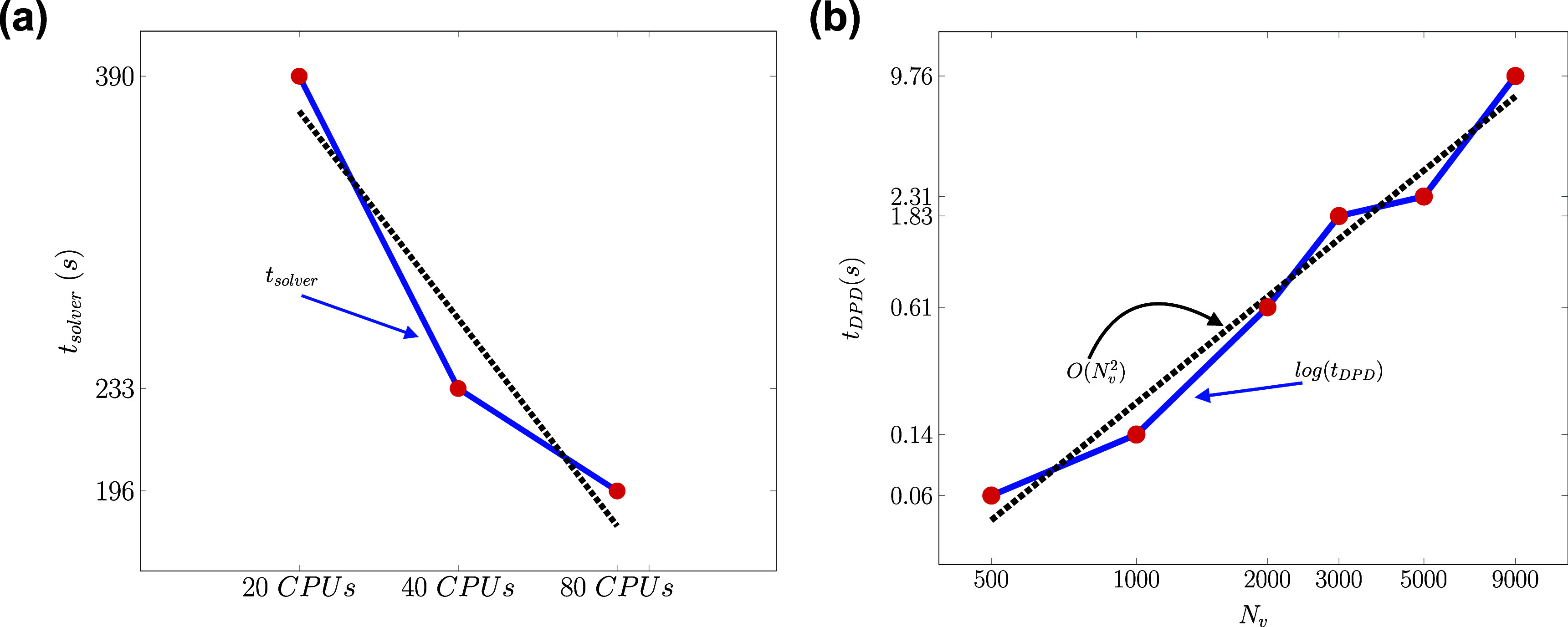
Scalability test. (a) Total wall-clock time (*t*_*solver*_) per time step for the solver to resolve the plasma flow and membrane dynamics using different computational resources (number of CPUs). Here, the computational configuration includes a RBC with *N*_*v*_ = 500, which is immersed in a plasma domain with 8 × 10^6^ grid points (Channel-4, Table III). The dashed line shows the linear scaling. (b) The performance of the DPD solver with respect to (*N*_*v*_ = 500, 1000, 2000, 3000, 5000 and 9000). The computational configuration is Channel-1 (Table III). The wall-clock time of the DPD solver (*t*_*DPD*_) for one simulation time step is plotted against *N*_*v*_ in the log–log scale. The dashed line represents the $O\left(N_{v}^{2}\right)$ scaling.

**FIG. 14.**
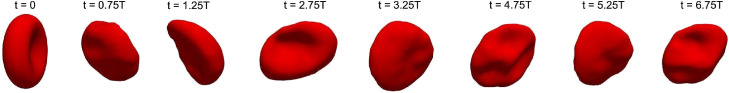
Morphology changes of the RBC under asymmetric sinusoidal waveform *I*_1_*U*_1_*r*_2_*χ*_1_. The shape transitions were recorded over an extended period of seven cycles (7T).

**FIG. 15.**
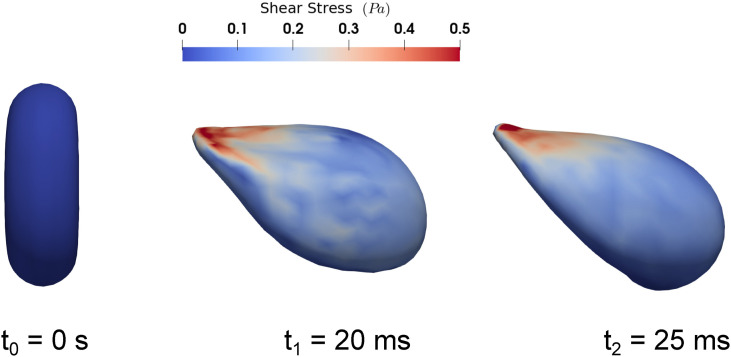
Loading condition on the case *I*_0_*U*_4_*r*_3_*χ*_1_ (slipper shape) over three time instances *t*_0_ = 0 s, *t*_1_ = 20 ms, and *t*_2_ = 25 ms.

## Data Availability

Data available on request from the authors.

## APPENDIX A: MATHEMATICAL FORMULATION OF DISSIPATIVE PARTICLE DYNAMICS

### 1. RBC membrane model—Γ_*RBC*_

In this work, the dissipative particle dynamics (DPD) methodologies for the membrane mechanics follow closely the previous studies in the literature.^30–33^

#### a. Action potential models

The viscoelasticity property of the membrane is modeled by a network of non-linear springs. The wormlike chain (WLC) spring accounts for the attractive force while the repulsive force is defined by the power (POW) spring. The combination of these springs is denoted as the WLC-POW model, which results in a system of non-linear springs with a non-zero equilibrium length,^30^ as discussed in the following.

The in-plane free energy term *V*_*in*–*plane*_ includes the elastic energy stored in the membrane modeled using the nonlinear Wormlike Chain and Power (*WLC*–*POW*) spring model. Here, the *WLC*–*POW* potential is computed for each link *j* formed by two vertices as

$$V_{in-plane}=\underset{j\in 1\;\ldots N_{s}}{\sum }\left[U_{\mathrm{WLC}}\left(l_{j}\right)+U_{\mathrm{POW}}\left(l_{j}\right)\right],$$ (A1)

with *N*_*s*_ being the total number of links forming the triangulated mesh.

The *WLC* attractive potentials *U*_*WLC*_(*l*_*j*_) for individual link *l*_*j*_ is expressed as

$$U_{\mathrm{WLC}}=\frac{k_{B}Tl_{\max}}{4p}\frac{3x^{2}-2x^{3}}{1-x},$$ (A2)

where the value $x=\frac{l_{j}}{l_{\max}}$ represents the spring deformation, in which *l*_*j*_, *l*_max_, *p*, *k*_*B*_, and *T* are the length of the spring *j*, the maximum allowable length for the links, the persistence length, Boltzmann’s constant, and the temperature, respectively.

The repulsive force, described by the energy potential *U*_*POW*_(*l*_*j*_), takes the form of a power function (*POW*). The separation distance *l*_*j*_ is a determining factor in the calculation of *U*_*POW*_, which is given by

$$U_{\mathrm{POW}}\left(l_{j}\right)=\frac{k_{p}}{\left(m-1\right)l_{j}^{m-1}}\;\;\;m>0\text{ and }m\neq 1,$$ (A3)

where *k*_*p*_ is the *POW* force coefficient. In this work, the value *m* = 2 is used for the exponent.^30^

The bending energy *V*_*bending*_ accounting for the membrane resistance to bending is defined as

$$V_{\mathrm{bending}}=\underset{j\in 1\;\ldots N_{s}}{\sum }k_{b}\left[1-\cos\left(\theta_{j}-\theta_{0}\right)\right],$$ (A4)

with *k*_*b*_, *θ*_0_, and *θ*_*j*_ being the bending rigidity, the spontaneous angle, and the instantaneous angle between the normal vectors of two adjacent triangles sharing a common edge (link) *j*, respectively.

The area and volume conservation constraints account for the incompressibility of the lipid bilayer and the inner cytosol, respectively. They are defined as

$$\begin{array}{c} \;V_{\mathrm{area}}=V_{\mathrm{area}}^{g}+V_{\mathrm{area}}^{\mathrm{loc}}=\frac{k_{a}{\left(A-A_{0}^{\mathrm{tot}}\right)}^{2}}{2A_{0}^{\mathrm{tot}}}+\underset{k\in 1\;\ldots N_{t}}{\sum }\frac{k_{d}\left(A_{k}-A_{0}\right)}{2A_{0}},\\ \;V_{\mathrm{volume}}=\frac{k_{v}{\left(V-V_{0}^{\mathrm{tot}}\right)}^{2}}{2V_{0}^{\mathrm{tot}}}, \end{array}$$ (A5)

with *N*_*t*_ being the total number of triangles. *k*_*a*_, *k*_*d*_, and *k*_*v*_ are the global area, local area, and volume constraint coefficients, respectively. *A*_*k*_ and *A*_0_ are the instantaneous area of the *k*th triangle (element) and the initial value of the average area per element. $A_{0}^{\mathrm{tot}}$ and $V_{0}^{\mathrm{tot}}$ are the RBC’s equilibrium total area and volume, respectively. *A* and *V* are the instantaneous total area and total volume of the RBC. The detailed procedure to evaluate the values of *A* and *V* for individual elements was reported in our previous work.^29^

Equation (2) is used to calculate the precise nodal forces for each potential energy *V* in Eqs. (A1)–(A5).^29,30^ The internal force $F_{i}^{\mathrm{membrane}}$ contribution from the *i*th vertex can be computed by summing all the nodal forces as

$$F_{i}^{\mathrm{membrane}}=F_{i}^{\mathrm{WLC}}+F_{i}^{\mathrm{POW}}+F_{i}^{\mathrm{Bending}}+F_{i}^{Area_{g}}+F_{i}^{Area_{\mathrm{loc}}}+F_{i}^{\mathrm{V olume}}.$$ (A6)

#### b. Membrane elastic properties

As the membrane mechanical response is modeled by Eq. (2), it is necessary to derive its elastic properties since they do not explicitly appear in the governing equations.^34^ Linear analysis^30,33^ on the non-linear spring network leads to a relationship between the macroscopic elastic properties (shear, area-compression, and Young’s moduli) and the model parameters. The derived linear shear modulus (*μ*_0_) of the WLC-POW non-linear model is

$$\mu_{0}=\frac{\sqrt{3}k_{B}T}{4pl_{\max}x_{0}}\left[\frac{x_{0}}{2{\left(1-x_{0}\right)}^{3}}-\frac{1}{4{\left(1-x_{0}\right)}^{2}}+\frac{1}{4}\right],$$ (A7)

where *l*_0_ is the equilibrium spring length and $x_{0}=\frac{l_{0}}{l_{\max}}$. The linear area-compression modulus (*K*) and Young’s modulus (*Y*) are

$$K=2\mu_{0}+k_{a}+k_{d},$$ (A8)

$$Y=\frac{4K\mu_{0}}{K+\mu_{0}}.$$ (A9)

Due to the incompressibility of the membrane *μ*_0_ ≪ *k*_*a*_ + *k*_*d*_, it can be shown that *Y* ≈ 4*μ*_0_. In addition, the model bending coefficient *k*_*b*_ and the macroscopic membrane bending rigidity *k*_*c*_^30,33,35^ are related as $k_{b}=\frac{2k_{c}}{\sqrt{3}}$.

### 2. Modeling the interaction between RBC’s membrane (Γ_*RBC*_) and the cytosol fluid (Ω_*c*_)

The mathematical formulation of the conservative force $F_{ij}^{C}$, the dissipative force $F_{ij}^{D}$, the random force $F_{ij}^{R}$, and the penalty force $F_{ij}^{P}$ for the membrane and the cytosol fluid particles are explained in the following.

#### a. The conservative force

The conservative force $F_{ij}^{C}$ is given by:

$$\begin{array}{c} F_{ij}^{C}=F_{ij}^{C}\left(r_{ij}\right){\hat{r}}_{ij},\\ F^{C}\left(r_{ij}\right)=\left\{\begin{array}{cc} a_{ij}\left(1-\frac{r_{ij}}{r_{c}}\right)\; & \;\;\text{for}\;\;r_{ij}\leq \;r_{c},\\ 0\; & \;\;\text{for}\;\;r_{ij}>\;r_{c}, \end{array}\right. \end{array}$$ (A10)

where *a*_*ij*_ = 20 is the conservative force coefficient between particles *i* and *j*. Note that the particles *i* and *j* can be either membrane or cytosol fluid particles. Thus, there are two types of interactions: i) cytosol fluid/fluid and ii) membrane/fluid–particle interactions.^30^ The cutoff radius *r*_*c*_ = 1 is set for all simulations. The summary of force strength coefficients^68^ are shown in Table XII.

**TABLE XII.** Force strength coefficients for DPD interactions.

Interaction	*a*_*ij*_	*γ*	*r*_*c*_
Fluid-fluid	20	116.4	1
Membrane-fluid	20	116.4	1

#### b. The dissipative force

To account for the viscous contribution in the RBC membrane (**F**^**membrane**^), a dissipative force is incorporated by modifying the spring definition. The dissipative force $F_{ij}^{D}$ is computed for each spring, where *i*, *j* ∈ [1 … *N*_*v*_] are a pair of two network vertices, which are connected by a link (spring). The dissipative force $F_{ij}^{D}$ for the membrane particles is computed as

$$F_{ij}^{D}=-\Gamma^{T}v_{ij}-\Gamma^{C}\left(v_{ij}\cdot {\hat{r}}_{ij}\right){\hat{r}}_{ij}.$$ (A11)

The membrane viscosity is a function of both dissipative parameters, Γ^*T*^ and Γ^*C*^. The superscripts *T* and *C* denote the translational and central components. Here, Γ^*T*^ is responsible for a large portion of the membrane viscosity in comparison to Γ^*C*^. In addition, Γ^*C*^ is assumed to be equal to one-third of Γ^*T*^ in Eq. (A11).^30^ Consequently, these parameters relate to the physical viscosity of the membrane *η*_*m*_ as

$$\left\{\begin{array}{cc} \eta_{m}=\sqrt{3}\Gamma^{T} & +\frac{\sqrt{3}\Gamma^{C}}{4},\\ \Gamma^{C}=\frac{\Gamma^{T}}{3}. \end{array}\right.$$ (A12)

Hence, the dissipative force $F_{ij}^{D}$ among the membrane particles can be expressed as

$$F_{ij}^{D}=-\frac{12}{13\sqrt{3}}\eta_{m}v_{ij}-\frac{4}{13\sqrt{3}}\eta_{m}\left(v_{ij}\cdot {\hat{r}}_{ij}\right){\hat{r}}_{ij}.$$ (A13)

The dissipative force $F_{ij}^{D}$ for the cytosol fluid particles is defined as

$$F_{ij}^{D}=-\gamma \omega^{D}\left(r_{ij}\right)\left(v_{ij}\cdot {\hat{r}}_{ij}\right){\hat{r}}_{ij},$$ (A14)

where the quantity *γ* is a constant coefficient defining the strength of the dissipative force. The weight functions, *ω*^*D*^(*r*_*ij*_) and *ω*^*R*^(*r*_*ij*_), are given by

$$\omega^{D}\left(r_{ij}\right)={\left[\omega^{R}\left(r_{ij}\right)\right]}^{2},$$ (A15)

$$\omega^{R}\left(r_{ij}\right)=\left\{\begin{array}{cc} {\left(1-\frac{r_{ij}}{r_{c}}\right)}^{s}\; & \;\;\text{for}\;\;r_{ij}\leq \;r_{c},\\ 0\; & \;\;\text{for}\;\;r_{ij}>\;r_{c}, \end{array}\right.$$ (A16)

with *s* = 1 following the original DPD method.^30^ The particle *i* represents the fluid particle, while the particle *j* can be either a fluid or membrane particle within the cutoff radius *r*_*c*_.

#### c. The random force

The random force $F_{ij}^{R}$ in Eq. (A17) is defined following the general framework of the fluid particle model^30^ and using the assumption of the membrane viscosity in Eq. (A12),

$$F_{ij}^{R}dt=\sqrt{2k_{B}T}\left(2\sqrt{\frac{2\sqrt{3}}{13}\eta_{m}}\;d\bar{W_{ij}^{S}}\right){\hat{r}}_{ij},$$ (A17)

where *dt* is the physical time step, *tr*(*d***W**_*ij*_) is the trace of the random matrix of independent Wiener increments *d***W**_*ij*_, and $d\bar{W_{ij}^{S}}=dW_{ij}^{S}-\frac{tr\left(dW_{ij}^{S}\right)}{3}$ is the traceless symmetric part.

The random force $F_{ij}^{R}$ for the cytosol fluid is defined as

$$F_{ij}^{R}=\sigma \omega^{R}\left(r_{ij}\right)\cdot \frac{\vartheta_{ij}}{\sqrt{dt}}\cdot {\hat{r}}_{ij},\;\;\;\sigma^{2}=2\gamma k_{B}T,$$ (A18)

where *σ* is a constant coefficient defining the strength of the random force, *ϑ* is a normally distributed random variable with zero mean and unit variance, and *ϑ*_*ij*_ = *ϑ*_*ji*_. Note that the particles *i* and *j* must be both cytosol fluid particles.

#### d. Plasma and cytosol viscosity contrast

Under the physiological condition, the viscosity ratio between the blood plasma and the RBC cytosol is equal to 5.0 $\left(\lambda =\frac{\mu_{\mathrm{cytosol}}}{\mu_{\mathrm{plasma}}}=5.0\right)$.^69^ To ensure that this condition is met, the dynamic viscosity of the plasma is set to be *μ*_*plasma*_ = 1.2 mPa s.^70–72^ The viscosity condition is enforced on the cytosol fluid by calibrating the parameters of the dissipative and the random forces^73^ (*γ* and *σ*). In particular, the dynamic properties of the DPD particles of the cytosol fluid are given in the dimensionless DPD unit as^74^

$$\begin{array}{c} \text{mass diffusivity: }D_{f}=\frac{45k_{B}T}{2\pi \gamma \rho r_{c}^{3}},\\ \text{dynamics viscosity: }\mu =\frac{\rho D_{f}}{2}+\frac{2\pi \gamma \rho^{2}r_{c}^{5}}{1575}, \end{array}$$ (A19)

with *ρ* being the density. The DPD dimensionless parameters and physical units are linked^75^ in order to compute the coefficients of the dissipative and randoms forces for the cytosol dynamic viscosity of *μ*_*cytosol*_ = 6 mPa s. The details of the conversion procedure are summarized in Table XIII.

**TABLE XIII.** Relationship between DPD parameters and the physical units for viscosity ratio *λ* = 5. *r*_*c*_, *δt*, *μ*_*cytosol*_, and *γ* correspond to the cutoff radius, time step, dynamic viscosity of the cytosol, and coefficient defining the strength of the dissipative force, respectively. The definitions of the parameters *r*_*M*_, *τ*, *η*, and *η*_*M*_ are explained in Table I and sections of Appendixes A 2 d and A 3.

Parameters	DPD value	Scaling value	Physical value
*r*_*c*_	1	*r*_*M*_	9.7 × 10^−7^ m
*δt*	10^−3^	*τ*	2.5 × 10^−4^ s
*μ*_*cytosol*_(*γ* = 10)	0.5	$\frac{\eta }{\eta_{M}}$	6 × 10^−3^ Pa s

### 3. Scaling of model and physical units

One challenge in DPD modeling is establishing a relationship between the modeled quantities and the physical values. Since this relationship is not explicit, it is necessary to use a scaling argument to recover this relationship.^30,36^ For each parameter, the superscript *M* and *P* correspond to the model and physical units, respectively. The details of the physical parameters for the RBC are shown in Table I.

The length scale *r*^*M*^ is defined as

$$r^{M}=\frac{D_{0}^{P}}{D_{0}^{M}}\;\left(m\right),$$ (A20)

The energy per unit mass *k*_*B*_*T* and the force *N* scaling values are given by

$$\begin{array}{c} {\left(k_{B}T\right)}^{M}=\frac{Y^{P}}{Y^{M}}{\left(\frac{D_{0}^{P}}{D_{0}^{M}}\right)}^{2}{\left(k_{B}T\right)}^{P},\\ N^{M}=\frac{Y^{P}}{Y^{M}}\frac{D_{0}^{P}}{D_{0}^{M}}N^{P}, \end{array}$$ (A21)

with *Y* being the membrane of Young’s modulus.

The timescale *τ* is defined as follows:

$$\tau ={\left(\frac{D_{0}^{P}}{D_{0}^{M}}\frac{\eta_{m}^{P}}{\eta_{m}^{M}}\frac{Y^{M}}{Y^{P}}\right)}^{\alpha },$$ (A22)

with *α* = 1 being the scaling exponent.

### 4. Coarse-graining procedure

A full-scale model of the RBC typically consists of millions of particles, which are required to accurately simulate protein dynamics.^76^ However, it is not feasible to use such a full-scale model in a fluid–structure interaction (FSI) simulation due to the high computational cost. We followed the coarse-graining procedure of Ref. 31 to represent the RBC membrane by a smaller number of particles (coarse-grained model). This procedure does not allow a detailed simulation of separate proteins, but it is versatile enough to capture the overall dynamics of the RBC membrane. The parameters of the coarse-grained model (*c*) are computed from the ones of the fine-scaled model (*f*) by using a scaling procedure. Examples of such parameters are explained in the following.

Based on the equilibrium condition,^31^ a coarse-graining procedure is proposed based on the area/volume constraint for the spring equilibrium *l*_0_ and maximum *l*_max_ lengths as follows:

$$l_{0}^{c}=l_{0}^{f}\sqrt{\frac{N_{v}^{f}-2}{N_{v}^{c}-2}}\;\text{and}\;l_{\max}^{c}=l_{\max}^{f}\sqrt{\frac{N_{v}^{f}-2}{N_{v}^{c}-2}},$$ (A23)

where the role of *l*_0_ and *l*_max_ is critical in determining the response from the WLC model, as seen in Eq. (A2), with *l*_max_ = 2.2 *l*_0_ in the fine-scaled model. Due to the scaling in Eq. (A23), the value of $x_{0}=\frac{l_{0}}{l_{\max}}=\frac{1}{2.2}$ does not change as the model is coarse-grained from the number of vertices $N_{v}^{f}$ to $N_{v}^{c}$. In this work, a range of $N_{v}^{c}$ values are considered as shown in Table II, with $N_{v}^{f}$ set to be 27 472.

Furthermore, as the number of vertices reduces, the average angle between the pairs of adjacent triangles increases. Therefore, the spontaneous angle *θ* is adjusted accordingly in the coarse-grained model as

$$\theta_{0}^{c}=\theta_{0}^{f}\frac{N_{v}^{f}}{N_{v}^{c}}\;\text{with}\;\theta_{0}^{f}=\arccos\left(\frac{\sqrt{3}\left(N_{v}^{f}-2\right)-5\pi }{\sqrt{3}\left(N_{v}^{f}-2\right)-3\pi }\right).$$ (A24)

To maintain the shear and area-compression moduli, the parameters *p* and *k*_*p*_ are adjusted as

$$p^{c}=p^{f}\frac{l_{0}^{f}}{l_{0}^{c}}\;\text{and}\;k_{p}^{c}=k_{p}^{f}{\left(\frac{l_{0}^{c}}{l_{0}^{f}}\right)}^{m+1}.$$ (A25)

The details of the parameters for the coarse-graining process are shown in Table II.

## APPENDIX B: COMPUTATIONAL COST

In this section, we report the scalability of our numerical algorithm. Two tests were performed.

• *Test 1*—*Strong scaling test*: 8 × 10^6^ grid points for the blood plasma domain (Channel-4 in Table III) is considered while the number of vertices for the triangulated mesh of the RBC is kept as *N*_*v*_ = 500 vertices. The purpose of this test is to investigate the scalability of the FSI coupling as the number of CPUs increases.
• *Test 2*—*DPD scaling test*: multiple resolutions of the RBC triangulated mesh are tested *N*_*v*_ = 500, 1000, 2000, 3000, 5000 and 9000 with a fixed computational domain of 1 × 10^6^ grid points (Channel-1 in Table III) for the blood plasma on a single CPU. The purpose of this test is to investigate the computational cost of the DPD algorithm as the number of particles increases.

Our FSI methodology closely follows a linear scaling in Test 1 when the number of CPUs is increased from 20 to 80, as shown in Fig. 13(a). This result indicates that our FSI algorithm utilizes the computing resources reasonably well. Furthermore, the dependence of the computational cost on the number of vertices (*N*_*v*_) is investigated in Fig. 13(b). In this Test 2, the result shows that the computational time for DPD algorithm scales with $O\left(N_{v}^{2}\right)$ , which is expected due to the pairwise interaction between particles. This result also justifies the need for the coarse-grained procedure in Appendix A 4 in order to reduce *N*_*v*_ while maintaining accurate dynamics of RBCs.

We carry out simulations for only two cycles (2*T*) in all cases as it is sufficient to identify the differences in the shape transformation of RBCs under different flow conditions. For future bio-engineering applications, it is necessary to perform simulations in many cycles. As shown in Fig. 14, the case *I*_1_*U*_1_*r*_2_*χ*_1_ is simulated in seven cycles. The RBC tends to repeat its morphological shape in each cycle (after the third cycle). However, its shape does not return exactly to the one in the previous cycle. Comparing the instances (for example, *t*_5_ = 3.25*T* and *t*_7_ = 5.25*T*), it is clear that the shape varies significantly from one cycle to another. Such variability needs to be investigated in future studies.

To demonstrate the distribution of loading condition on the surface of the RBC membrane, the magnitude of the external loading is plotted in Fig. 15 for the case *I*_0_*U*_4_*r*_3_*χ*_1_. Although the incoming flow is stationary, the loading condition changes over time as the RBC deforms. The non-uniform distribution of the applied load offers important insights into the mechanisms behind the RBC shape transitions. Therefore, further investigation of this phenomenon is needed in future work.
